# A Low Dose of Berberine Is Metabolized in Weaned Piglets Without Major Changes to Gut Morphology or Gut Microbiota

**DOI:** 10.3390/ani15162450

**Published:** 2025-08-21

**Authors:** Christina Mouchtoglou, Marc Cherlet, Tessa Dehau, Marijke Aluwe, Richard Ducatelle, Evy Goossens, Siska Croubels, Filip Van Immerseel

**Affiliations:** 1 Livestock Gut Health Team (LiGHT) Ghent, Department of Pathobiology, Pharmacology and Zoological Medicine, Faculty of Veterinary Medicine, Ghent University, 9820 Merelbeke, Belgium; 2 Laboratory of Pharmacology and Toxicology, Department of Pathobiology, Pharmacology and Zoological Medicine, Faculty of Veterinary Medicine, Ghent University, 9820 Merelbeke, Belgium; 3 Translational Research in Gastrointestinal Disorders (TARGID), Faculty of Medicine, Katholieke Universiteit Leuven, 3000 Leuven, Belgium; 4 Flanders Research Institute for Agriculture, Fisheries and Food (ILVO), 9090 Merelbeke, Belgium

**Keywords:** berberine, weaning, metabolites, gut health, UPLC-MS/MS

## Abstract

Weaning is one of the most challenging stages in a piglet’s life, with multiple stressors contributing to poor gut health. For several years, zinc oxide (ZnO) was the preferred means of promoting a healthy gut and preventing post-weaning diarrhea (PWD). However, with the banning of its use at medicinal levels in the EU since 2022, alternatives are needed. Berberine (BBR), an isoquinoline alkaloid, has been used for centuries in Chinese medicine to treat diarrhea and has pharmacological properties that could make this molecule an attractive alternative to ZnO. The aim of this study was to investigate how berberine is metabolized in the intestinal tract and liver of weaned piglets; determine which metabolites are detected in intestinal contents and plasma; and whether a low dose can alter histomorphological parameters, short-chain fatty acid (SCFA) production, and gut microbiota composition. A total of 60 piglets weaned at 4 weeks were divided into two groups (Control and BBR), each consisting of six pens of five animals. After two weeks of feeding with a normal diet or a berberine-supplemented diet (30 mg berberine/kg feed), berberine and its metabolites were quantified in intestinal contents and plasma by ultra-performance liquid chromatography–tandem mass spectrometry (UPLC-MS/MS) on 12 animals per group (2 male piglets per pen). Moreover, villus length and crypt depth were measured in small-intestinal tissue, and 16S rRNA gene sequencing was performed to examine jejunal, cecal, and colonic gut microbiota composition. Our findings show that piglets metabolize berberine into phase I and II metabolites; however, a low dose does not affect their histomorphology, microbiota composition, or SCFA production.

## Simple Summary

Berberine is a compound found in plants and has been used in Chinese medicine for centuries to treat diarrhea. Its pharmacological properties have been shown to promote gut health. In this study, we evaluated how berberine, added at a low dose in feed for 2 weeks, is metabolized and affects the intestinal health of weaned piglets by quantifying berberine and its main metabolites in plasma and intestinal contents from all segments of the gastrointestinal tract. Additionally, we investigated whether a low dose of berberine is able to influence small-intestinal morphological parameters, short-chain fatty acid (SCFA) production, and gut microbiota composition. These results provide insight into how berberine is metabolized in piglets but also show that a low dose is insufficient to promote beneficial effects in the gut of unchallenged piglets.

## 1. Introduction

The EU is the second-biggest pork producer in the world after China and one of the biggest exporters. Amongst the different types of red meat, pork is the most widely consumed meat in the EU, with Germany, Spain, and France being the main producers. Farmers have to manage the increase in demand while maintaining animal health, as well as prevent economically impactful diseases such as post-weaning diarrhea (PWD), an important enteric disease that causes animals to produce watery discharges, become dehydrated, and lose weight. This leads to impaired growth and increased mortality, hence its negative economic and welfare impact. PWD is associated with the proliferation of enterotoxigenic *Escherichia coli* (ETEC), for which multiple predisposing factors have been described, including housing conditions, genetics, and young weaning age, that can impair the gut microbiome and gut barrier integrity and promote a leaky gut [1,2,3].

For years, zinc oxide (ZnO) has been added to piglet’s feed at medicinal levels of 2500–3000 ppm (mg/kg) as a measure to help prevent PWD due to its variety of beneficial effects; these include improving the intestinal morphology by increasing the villus-to-crypt ratio and tight-junction protein expression, a reduction in pro-inflammatory cytokine expression [4], the regulation of gut microbiota structure [5,6] and the protection of epithelial cells against damage caused by ETEC [7].

Since 2022, the EU has banned the use of medicinal levels of ZnO due to the risk of the (co)-selection of resistant bacteria and the environmental impact on plant growth and biodiversity due to accumulated Zn in the soil from the pig manure used in agriculture [8,9]. It is, therefore, important to find a compound to replace ZnO in promoting gut health in the post-weaning period.

Berberine (BBR) is an isoquinoline alkaloid used in Chinese medicine and can be found in plants such as *Coptis chinensis* and various *Berberis* species. Berberine shows beneficial effects in a variety of disorders by regulating gut microbiota, stimulating SCFA (short-chain fatty acid) production, and protecting the epithelial barrier from inflammatory responses, thereby promoting a healthy gut [10,11,12]. These gut-health-promoting properties make berberine an interesting alternative to ZnO for the prevention of PWD. However, evidence of the effects of berberine in weaned piglets is still scarce. Moderate inclusion levels (0.25–0.5 g berberine/kg feed) are generally associated with improved intestinal health in piglets in addition to reducing the expression of pro-inflammatory cytokines [13,14,15,16], while higher doses (1 g/kg feed) have been linked to less favorable effects in piglets and broilers, such as shifts in the microbiota structure to resemble a dysbiosis-like state [13,15,17]. Given these findings, we aimed to investigate whether a much lower dietary inclusion (0.03 g/kg feed) could elicit beneficial effects. Berberine is metabolized mainly in the large intestine (by both host and microbial enzymes) and the liver [18]. The main metabolic pathways include demethylation, demethylenation, and reduction, resulting in the following phase-I metabolites: demethyleneberberine, dihydroberberine, berberrubine and thalifendine, and columbamine and jatrorrhizine, with the last four being isomeric pairs. Phase I metabolites can undergo further metabolization in the liver by conjugation with glucuronic acid and sulfate to obtain phase II metabolites. It has been reported that these berberine-derived metabolites play a significant role in berberine’s gut-health-promoting effects in vivo [19]. However, the metabolism of berberine in pigs has not yet been investigated in vivo.

Given berberine’s potential to promote gut health, the aim of this study was to analyze how berberine is metabolized across intestinal segments in weaned piglets. Furthermore, we sought to investigate whether low-dose administration of berberine affects gut microbiota composition in the small and large intestine, as well as SCFA production in the large intestine, and whether it also induces gut morphological changes in the small intestine.

## 2. Materials and Methods

### 2.1. Experimental Design and Sample Collection

A total of 60 TN70 x Piétrain 4-week-old piglets from 8 different sows were provided by the Flanders Research Institute for Agriculture, Fisheries and Food (ILVO) (Melle, Belgium). At 4 weeks of age, the piglets were weaned and allocated to two experimental groups: Control (CTR) and BBR. Each group consisted of 6 pens that housed 5 piglets/pen. Piglets from each sow were equally divided over Control and BBR pens, ensuring a balanced distribution of piglets across the experimental conditions. The Control group was fed the basal diet (Table 1), while the BBR group was given the basal diet supplemented with berberine 0.03 g/kg feed. Berberine was given as berberine chloride purchased from Merck (Sigma-Aldrich, Hoeilaart, Belgium). It was incorporated in the pellets of the feed by using cold pelleting equipment [20]. The feed was provided *ad libitum* for the duration of the 2-week trial. Quantification of BBR in the pellets confirmed that the dose was 29.9 ± 2.4 mg BBR/kg feed, as described in Supplementary Methods S1.

Piglets were weighed at the start and end of the trial. After two weeks of feeding the control or berberine-supplemented diet, two male piglets per pen were selected for further analysis, resulting in a total of 24 animals originating from 6 different sows (Supplementary Table S1). The selection was based on their body weight at 28 days of age (CTR: 8.5 ± 0.6 kg, BBR: 8.9 ± 0.9 kg). All 24 piglets aged 6 weeks were anesthetized intramuscularly with a mixture of 0.15 mL/kg of zolazepam and tiletamine (Zoletil^®^ 100, Virbac, Leuven, Belgium) and xylazine (Xyl-M^®^ 2%, VMD, 0.15 mL/kg) before euthanizing them with 200 mg/mL of Pentobarbital Sodium (Vetorder Medini NV, Legeweg Oostkamp, Belgium).

After euthanasia at 6 weeks of age, blood was collected from all 24 animals in 5 mL heparin tubes, centrifuged at 3000 rpm for 10 min at 4 °C, and then stored at −20 °C until further analysis. In addition, intestinal contents from each of the 24 animals were collected from the duodenum, proximal/middle/distal jejunum, ileum, cecum, and colon for berberine quantification. 16S rRNA gene sequencing was performed on middle jejunum, cecum, and colon samples, while SCFAs were quantified from the cecum and colon. The content was snap-frozen immediately in liquid nitrogen and kept on dry ice until storage at −70 °C. Approximately 2 cm of tissue from each of the 24 piglets was obtained from the same small intestinal segments and stored in 4% phosphate-buffered formaldehyde for histological examination.

### 2.2. Intestinal Morphology

Intestinal tissue segments were fixed in 4% phosphate-buffered formaldehyde for 24 h before being embedded in paraffin wax and sectioned at 5 μm. The slides were stained with hematoxylin and eosin, followed by PC-based image analysis of the villus length and crypt depth using Leica Application Suite V4 (LAS V4; Leica, Diegem, Belgium). Measurements were taken from at least 7 randomly selected villi/crypts, and the average per animal was calculated.

### 2.3. DNA Extraction from Intestinal Contents

DNA was extracted from intestinal contents using the cetyltrimethylammonium bromide (CTAB) method [21]. First, 200 mg of jejunal or 100 mg of cecal or colonic contents was suspended in 500 μL of CTAB buffer (>98% (Sigma-Aldrich) 5% (wt/vol), 0.35 M NaCl, 120 nM K_2_HPO_4_) and 500 μL of phenol–chloroform–isoamyl alcohol (25:24:1). The mixture was homogenized by grinding (2×) with 500 mg of unwashed glass beads (Sigma-Aldrich) in a bead beater (1.5 min, 22.5 Hz; TissueLyser; Qiagen, Hilden, Germany) with a 30 s interval between shakings. Samples were centrifuged for 10 min at 8000 rpm, and 300 μL of the supernatant was transferred to a new tube. A second extraction from the remaining contents was performed by adding 250 μL of CTAB buffer and homogenizing and centrifuging the sample as described above. An equal volume (600 μL) of chloroform–isoamyl alcohol (24:1) was added to the supernatant collected to remove the phenol from the samples. The mixture was further centrifuged at 13,053 rpm for 10 s. A 500 μL aliquot of the aqueous phase was transferred to a new tube. Nucleic acids were precipitated with 2 volumes of PEG-6000 solution (polyethylene glycol 30% wt/vol; 1.6 M NaCl) for 2 h at room temperature. Samples were centrifuged (11,766 rpm, 20 min) and washed with 1 mL ice-cold ethanol (70% vol/vol). This step was repeated twice. The pellet was then obtained by centrifugation (11,766 rpm, 20 min), dried, and resuspended in 50 μL of deionized water (LiChrosolv Water; Merck, Darmstadt, Germany). The quality and the concentration of the DNA were examined spectrophotometrically (NanoDrop; Thermo Scientific, Waltham, MA, USA).

### 2.4. 16S rRNA Gene Sequencing and Bioinformatics

In order to perform a taxonomic characterization of jejunal, cecal, and colonic microbiota, the V3 to V4 hypervariable region of the 16S rRNA gene was amplified using the primers S-D-Bact-0341-b-S-17 (5′-CCTACGGGNGGCWGCAG-3′) and S-D-Bact-0785-a-A-21 (5′-GACTACHVGGGTATCTAATCC-3′) [22], as described by Aguirre et al., 2019 [21]. The final products were pooled at an equimolar concentration of 10 nM and sequenced with 30% PhiX spike-in using Illumina MiSeq v3 technology (2 × 300 bp, paired-end) at Macrogen (Amsterdam, The Netherlands).

Demultiplexing of the amplicon dataset and deletion of the barcodes were performed by the sequencing provider. All further processing was performed in R (v4.2.1) [23]. Raw sequence reads were trimmed, quality-filtered, and dereplicated using the DADA2 algorithm (v1.24.0) [24]. An initial amplicon sequence variant (ASV) table was constructed before chimeras were identified using the *removeBimeraDenovo* function. Finally, taxonomy was assigned using DADA2′s native naïve Bayesian classifier against the Silva database (v1.38) [25]. To construct a phylogenetic tree, multiple sequence alignment was performed using the DECIPHER (v2.24.0) algorithm [26], after which a neighbor-joining tree was constructed using phangorn (v2.10.0) [27]. This neighbor-joining tree was used as the starting point to fit the final GTR + G + I (generalized time-reversible with gamma rate variation) maximum likelihood tree. The resulting phylogenetic tree and ASV table were loaded into *phyloseq* (v1.42.0) [28], after which potential contaminant chloroplastic and mitochondrial ASVs were removed from the dataset.

### 2.5. SCFA Quantification

The amount of acetic acid, propionic acid, butyric acid, valeric acid, isobutyric acid, isovaleric acid, isocaproic acid, total branched-chain fatty acids (bCFAs), and total SCFAs in intestinal contents was quantified using gas chromatography at Cryptobiotix (Technologiepark-Zwijnaarde, Ghent, Belgium). In short, SCFAs were extracted from cecum and colon contents using diethyl ether after the addition of 2-methyl hexanoic acid as an internal standard. Extracts were analyzed using a GC-2014 gas chromatograph (Shimadzu, ‘s-Hertogenbosch, The Netherlands) equipped with a capillary fatty-acid free EC-1000 Econo-Cap column (Alltech, Laarne, Belgium), a flame ionization detector, and a split injector.

### 2.6. Berberine Quantification in Plasma and Small and Large Intestinal Contents

Berberine and its metabolites were quantified in plasma and intestinal contents of animals from the Control and BBR group using the method previously described and validated in Dehau et al., 2023 [17], with minor changes.

#### 2.6.1. Plasma Sample Preparation Procedure

A 137.5 µL plasma sample was used for sample extraction, which consists of 125 µL of plasma to which, after in vivo sampling, 12.5 µL of an ascorbic acid solution of 170 mg/mL in water was added, before storage at ≤−15 °C until UPLC-MS/MS analysis in a 1.5 mL Eppendorf cup. The addition of ascorbic acid solution was performed to prevent oxidation of dihydroberberine to berberine during sample storage (according to Feng et al., 2015) [29]. Subsequently, 50 µL of Milli-Q water and 25 µL of an internal standard solution containing 100 ng/mL of both berberine-d6 and tetrahydropalmatine in water were added. A 640 µL volume of 1% (*v*/*v*) formic acid solution in acetonitrile (1/3 ratio) was added, followed by thorough vortex mixing (1 min at 2500 rpm on a vortex instrument (Bench Mixer™ Multi Tube Vortexer, Benchmark Scientific, Sayreville, NJ, USA)). Thereafter, the sample was centrifuged at 13,000 rpm for 10 min. at 4 °C. The supernatant was transferred to an Ostro^TM^ 96-well plate (25 mg) (Waters, Milford, MA, USA). A vacuum was applied to the Ostro^TM^ plate for 5 min., and the eluate was collected in a 2 mL square 96-well collector plate. The sample was transferred to a glass tube and evaporated at 40 °C under a gentle stream of nitrogen in a Pierce Reacti-Therm III™ Heating Module and Reacti-Vap™ III Module (Rockford, IL, USA). The dried sample extract was reconstituted in 500 µL of Milli-Q water, followed by injection of a 5 µL sample aliquot onto the UPLC-MS/MS apparatus.

Calibrator and all other spiked samples (quality control samples) were prepared as follows: 125 µL of blank plasma (obtained from animals that were administered control feed, without berberine added) + 12.5 µL of ascorbic acid 170 mg/mL solution + 25 µL mixed working solution of berberine and metabolites (with the exception of dihydroberberine) in water at different levels + 25 µL of dihydroberberine working solution in water containing ascorbic acid at 17 mg/mL at different levels + 25 µL of an internal standard solution containing 100 ng/mL of both berberine-d6 and tetrahydropalmatine in water. The calibration curve was in the 0.1–100 ng/mL range and included the 0.1, 0.25, 0.5, 1, 2.5, 5, 10, 25, 50, and 100 ng/mL levels. Quantification was based on the ratio of the analyte peak area compared to the peak area of the internal standard berberine-d6 for all components, except dihydroberberine and oxyberberine, where tetrahydropalmatine was used as the internal standard.

#### 2.6.2. Intestinal Content Sample Preparation Procedure

A 0.25 g sample of contents from different intestinal segments was weighed in a 15 mL polypropylene centrifuge tube. For stabilization of dihydroberberine, 250 µL of ascorbic acid 170 mg/mL solution was added, followed by brief vortex mixing. Subsequently, 25 µL of an internal standard solution containing 2.5 µg/mL of both berberine-d6 and tetrahydropalmatine in water was added, followed by brief vortex mixing. A 2.5 mL volume of extraction solvent was then added, consisting of a 1% (*v*/*v*) formic acid solution in methanol. The sample was put on a rotary shaker set at 80 rpm for 20 min, followed by centrifugation (4000 rpm, 10 min, 4 °C). The supernatant was then diluted with a factor of 1/5, prepared directly in an autosampler vial as follows: 200 µL of sample extract +100 µL of ascorbic acid 170 mg/mL solution +700 µL of Milli-Q water, followed by vortex mixing (15 s at 2500 rpm on the vortex instrument). A 2.5 µL sample aliquot was injected onto the UPLC-MS/MS apparatus.

The calibrator and all other spiked samples (quality control samples) were prepared as follows: 0.25 g blank intestinal contents (obtained from animals that were administered control feed without berberine added) +250 µL of ascorbic acid 170 mg/mL solution +25 µL of mixed working solution of berberine and metabolites (with the exception of dihydroberberine) in water at different levels +25 µL of dihydroberberine working solution in water containing 17 mg/mL ascorbic acid at different levels + 5 µL of an internal standard solution containing 2.5 µg/mL of both berberine-d6 and tetrahydropalmatine in water. The calibration curve was in the 25–5000 ng/g range and included the 25, 50, 100, 250, 500, 1000, 2500, and 5000 ng/g levels. Quantification was based on the ratio of the analyte peak area compared to the peak area of the internal standard berberine-d6 for all components, except dihydroberberine and oxyberberine, where tetrahydropalmatine was used as the internal standard.

#### 2.6.3. Method Validation

The UPLC-MS/MS analysis method used for the quantification of berberine and berberine-derived metabolites both in plasma and intestinal contents was validated according to the guidelines of the European Medicines Agency, i.e., the Committee for Veterinary Medicinal Products (EMEA/CVMP/VICH/463202/2009) [30] and the Committee for Medicinal Products for Human Use (EMA/CHMP/ICH/172948/2019) [31], and in accordance with the EU Directive 2002/657/EC [32]. The following parameters were evaluated: linearity, within-day and between-day accuracy and precision, specificity, carry-over, and analyte stability in processed sample extract. The results of the method validation can be found in Supplementary Materials S2. Limits of quantification (LOQ) were 0.1 ng/mL for plasma and 5 ng/g for intestinal contents for all compounds, with the exception of dihydroberberine, where the LOQ in intestinal contents was established at 10 ng/g.

### 2.7. Statistics

Statistical analysis of body weight and morphological parameters was performed using GraphPad Prism (Version 8.4.3, San Diego, CA, USA) using an unpaired *t*-test or Mann–Whitney if the data did not pass the normality test. All other analyses were performed in R (v4.2.1).

Given the known lasting effect of the sow on the piglet’s microbiome, the identity of the sow (SowID) was included as either a random effect or confounder as appropriate for the analysis of SCFAs, microbiota composition, and berberine quantification [33,34]. The microbial alpha diversity (Chao1 richness) was calculated using *phyloseq* [28]. Differences in richness were assessed using linear mixed models using the *lme4* and *lmerTest* packages. Prior to beta diversity analysis, the ASV counts were transformed to proportions. The Bray–Curtis distance was used as a measure for the microbial beta diversity. The dispersion (variance) in the beta diversity was calculated using the *betadisper* function in the *vegan* package [35]. Differences in variance between groups were tested using ANOVA. Significant differences in the community composition between groups were determined through a permutational multivariate analysis of variance using distance matrices (PERMANOVA), using the *adonis2* function in *vegan*. Differentially abundant taxa (phyla, families, or genera) in the jejunal, colonic, or cecal microbiome between the control or BBR group were identified by applying *DESeq2* on the non-rarefied community composition data [36]. Significant differences were obtained using a Wald test followed by a Benjamini–Hochberg multiple hypothesis correction.

Differences in SCFAs, berberine, and metabolite quantification data were analyzed using linear mixed models using the *lme4* and *lmer Test* packages, followed by a Tukey post hoc test. BBR and metabolite levels were log-transformed. The model included the intestinal compartments as ordered factors (from proximal to distal). Additionally, different models were fitted for phase I and phase II metabolites to assess the differences in either phase I or phase II metabolite levels within each intestinal segment.

## 3. Results and Discussion

### 3.1. Low Dose of Berberine Does Not Affect Body Weight or Gut Morphology

In this study, we evaluated whether a low dose of berberine (0.03 g/kg), fed to 4-week-old piglets for two weeks, had any effect on body weight and gut morphological parameters. By the end of the trial, there was no difference in bodyweight (CTR: 11.2 kg ± 1.1, BBR: 11.3 kg ± 1.4, *p* = 0.803) (Table 2) between the two groups. Furthermore, we determined whether berberine could improve villus height, crypt depth, and villus-to-crypt ratio. Intestinal villi play a crucial role in the absorption of nutrients in the gut. An increase in the height of these villi signifies a larger surface area and enhanced absorption capacity. Intestinal crypts are located at the base of the villi and provide new cells for the regeneration of the villi. After weaning, the balance between the proliferation and apoptosis of intestinal epithelial cells is disrupted, resulting in the atrophy of intestinal villi and increased crypt cell proliferation.

We measured each of these parameters in the duodenum; proximal, middle, and distal jejunum; and ileum. No statistically significant differences in villus height between the two groups were found in any of the segments. Duodenal and ileal crypt depth were significantly increased in the BBR group. Only the proximal jejunum villus-to-crypt ratio was significantly decreased in the BBR group (Table 2). The increase in crypt depth without a corresponding reduction in villus height suggests an upregulation of epithelial cell proliferation, potentially as an adaptive response to berberine supplementation. This may reflect enhanced mucosal renewal rather than villus atrophy, as the maintenance of villus height indicates that absorptive capacity was not compromised. Du et al., 2025 [13] showed that ETEC-challenged piglets supplemented with a moderate dose of berberine (0.5 g/kg feed) exhibited higher villus height and villus-to-crypt ratio in the jejunum and lower villus height and crypt depth in the ileum compared to the ETEC group. In contrast, a higher dose (1 g/kg feed) showed opposite effects [13,15]. Another study showed that weaned piglets supplemented with a moderate dose of berberine (0.25 g/kg) had an increased villus height and villus-to-crypt ratio in the ileum [14]. This suggests that the effects of berberine on gut morphology may depend on the administration time, the intestinal segment, and the dose.

### 3.2. Microbiome Composition Is Not Significantly Altered with a Low Dose of Berberine

To investigate whether berberine affected the microbial composition of piglets, gut microbiota from contents of the mid jejunum, cecum, and colon were analyzed using 16S rRNA gene sequencing. Alpha diversity was measured, and the estimated species richness (Chao1 index) was reported (Table 3). In the mid jejunum, the Chao1 index was 115.72 (±102.39) for the BBR group, whereas it was 122.73 (±107.41) for the Control group, with no significant difference (*p* = 0.87). Similarly, no differences (*p* = 0.16) were observed in the cecum, with Chao1 for the BBR group being 381.8 (±92.3) and 331.6 (±70.7) for the Control group. Finally, no significant difference (*p* = 0.27) was reported in the colon, with a Chao1 index of 573.84 (±119.9) for the BBR group and 526.79 (±92.49) for the Control group.

The Bray–Curtis test was used to compare beta diversity between the Control group and piglets fed the BBR diet. No effect on microbial community composition was observed in any of the intestinal segments (mid jejunum: R^2^ = 2.3%, *p* = 0.876; cecum: R^2^ = 4%, *p* = 0.531; colon: R^2^ = 3.8%, *p* = 0.466). In comparison, there was a significant influence of the sow on the piglet microbiome in the colon, with SowID explaining 30% of the variation between the samples (R^2^ = 30%, *p* = 0.001) (Supplementary Figure S1). Tancredi et al., 2025 recently showed the effect of the sow on the piglets’ microbiome [34], while Lim et al., 2023 revealed that in 1-day-old piglets, sow feces contributed 6% to the piglet microbiome, and by the age of 28 days, that percentage was increased to 10% [37]. Overall, berberine did not cause a shift in microbiota diversity.

In order to evaluate whether there were any differentially abundant bacterial taxa between Control and BBR groups in the mid jejunum, cecum, and colon, DeSeq2 analysis was performed at the phylum, family, and genus levels. In the mid jejunum, no differences between the Control and BBR group were observed at the phylum level or family level. The only difference between Control and BBR-fed piglets was found at the genus level in the low-abundance genus *Latilactobacillus*, which had a relative abundance of 0.633% in Control piglets, but was not detected in the BBR-treated piglets. In the distal intestine, berberine tended to decrease the phylum *Actinobacteriota* (CTR: 1.106%, BBR: 0.688%; *p* = 0.007, *padj* = 0.07), while in the cecum, it tended to increase the phylum *Campylobacterota* (CTR: 0.081%, BBR: 0.601%; *p* = 0.01, *padj* = 0.07). At the genus level in the cecum, *Syntrophococcus* had a relative abundance of 0.139% in the Control group but was not detected in BBR-fed piglets. In the colon, the phylum *Proteobacteria* was higher in the BBR group (CTR: 0.664%, BBR: 2.447%; *p* = 0.004, *padj* = 0.05). At the genus level, *Syntrophococcus* had a very low relative abundance in the BBR group (CTR: 0.113%, BBR: 0.001%; *p* < 0.001, *padj* = 0.02). Overall, the effects of low-dose berberine (0.03 g/kg) on the abundance of specific taxa were limited. Berberine is known to alter the gut microbiota composition [10]; however, there is a lot of variation between animal species, doses used, etc. Increased *Proteobacteria* has been previously observed in the small and large intestine of broilers fed a high dose of berberine (1 g/kg), parallel to a positive effect on the small intestinal gut morphology [17]. The observed decrease in *Latilactobacillus*, *Syntrophococcus*, and *Actinobacteria*—all primarily Gram-positive bacteria—may reflect the mild antibacterial properties of berberine against sensitive bacteria [38,39].

Several studies investigated the effect of berberine on the gut microbiota of weaned or ETEC-challenged weaned piglets [13,14,15,16]. These studies clearly show a dose-dependent effect of berberine. The gut microbiota composition of ETEC-challenged piglets fed a high dose of berberine (1 g/kg feed) looked similar to that of ETEC-challenged piglets on a control diet, suggesting that higher doses of berberine may not be favorable. A dysbiosis-like microbiota was previously observed in broilers supplemented with 1 g BBR/kg feed [17]. When a more moderate berberine concentration was given to piglets (e.g., 0.25 g/kg or 0.5 g/kg), berberine seemed to positively impact the microbiome. For example, in piglets fed 0.25 g BBR/kg feed, the beta diversity index was significantly increased in the ileum compared to the control [14]. With a similar dose, Nie et al., 2024 showed that beta diversity in the cecum was higher in the BBR group compared to the control, and the two groups clustered separately in both colon and cecum [16]. Furthermore, a moderate dose of berberine (0.25 g/kg or 0.5 g/kg) fed to ETEC-challenged weaned piglets has been shown to promote beneficial bacteria (e.g., *Weissella*) while reducing those that are prominent after weaning (e.g., *Prevotella*) [13,14,15]. *Prevotella* levels are known to increase after weaning due to their role in non-starch polysaccharide fermentation [40]. Additionally, it has been identified as a key genus in maintaining balance with *Escherichia,* where a disrupted relationship may lead to the onset of diarrhea in weaned piglets [41]. The increase of beneficial bacteria following berberine intervention has been consistently reported in other animal species [11]. These favorable berberine-mediated shifts in the microbiota composition suggest that berberine may be a suitable supplement during weaning. However, similar shifts were not observed in our study. The dose used in this study was 8 to 16 times lower than the previously mentioned moderate doses (0.25 g/kg or 0.5 g/kg), in addition to a reduced administration period of berberine (2 weeks versus 17 or 21 days), consequently exposing the compound for a shorter period of time to the commensal bacteria, which may explain the limited effects observed on the gut microbiota.

### 3.3. Low Dose of Berberine Does Not Induce Changes in SCFA Production

SCFAs are produced by fermentation of indigestible polysaccharides by the gut microbiota in the large intestine [42,43]. The composition of the microbiota can influence the availability of SCFAs, which are important energy contributors in pigs. For example, butyrate is the main energy source for colonocytes [43,44]. In addition, SCFAs play a key role in maintaining intestinal gut barrier function and reducing inflammation and oxidative stress [44,45,46], all of which are compromised during weaning.

For this reason, SCFAs were measured in the cecum and colon contents from piglets in the Control and BBR groups. Acetic acid was the most abundant SCFA, followed by propionic and butyric acid, in both cecal and colonic contents. When comparing the two groups, no differences were observed in individual or total SCFAs in both the cecum and colon (Table 4). While berberine was previously shown to increase the abundance of SCFA-producing bacteria in the large intestine of weaned piglets challenged or not with ETEC, associated effects on luminal SCFA concentrations have so far not been reported [13,15,47]. This study is the first to present data on the effects of berberine on SCFA production in weaned piglets. One study investigating the combined effect of a moderate dose of berberine (10 mg/kg of bodyweight) and ellagic acid (10 mg/kg of bodyweight) in weaned piglets showed that these animals had increased propionate and butyrate concentrations in the jejunum and colon [48]. Increased SCFA production after berberine administration has been reported in other animal species [11]. The limited changes in gut microbiota composition observed in this study may explain why a low dose of berberine did not impact SCFA production. Further studies are needed to investigate whether a higher dose of berberine can stimulate SCFA-producing bacteria and/or SCFA pathways, as well as SCFA concentrations in the intestine.

### 3.4. Berberine Quantification in Plasma and Intestinal Contents of Piglets

Berberine has a very low oral bioavailability, resulting in low plasma concentrations and accumulation in the intestinal lumen [49,50,51]. It is poorly absorbed by the intestinal epithelium and is extensively metabolized to phase I metabolites by gut microbial and host enzymes. Therefore, only a small amount of berberine reaches the liver along with phase I metabolites, where the latter undergo phase II metabolization to obtain glucuronide or sulfate conjugates. From there, the metabolites are either distributed to other tissues, re-enter the gut lumen via the bile, or are excreted in the urine [19,29]. In the intestinal lumen, the gut microbiota can deconjugate phase II metabolites that have re-entered, hence once more generating phase I metabolites.

The quantification of berberine and its metabolites was performed using UPLC-MS/MS in samples from piglets fed a berberine-supplemented diet. Additionally, Figures S2–S4, as well as the Supplementary Tables S2–S3, show all results obtained. It should be mentioned that levels below the LOQ must be considered as indicative levels.

**Figure 1:**
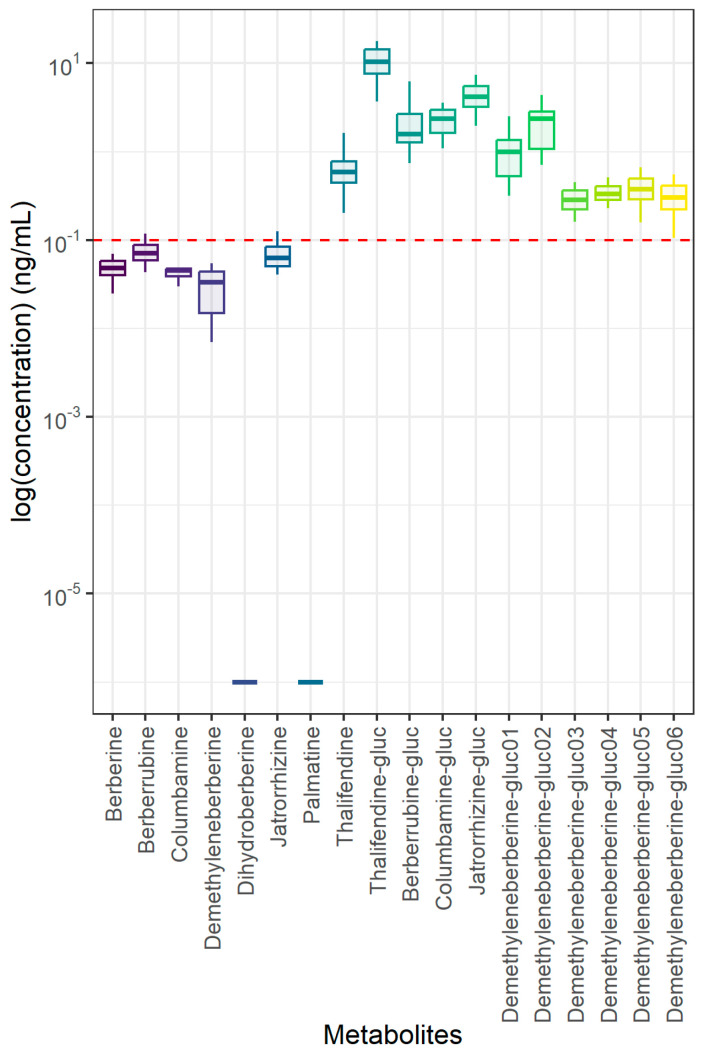
Berberine and phase I and II metabolites in plasma of 6-week-old piglets fed 30 mg BBR/kg feed for 2 weeks (n = 12). Red dotted line represents the limit of quantification (LOQ) established at 0.1 ng/mL during method validation experiments, while whiskers represent the range within 1.5 × IQR from the quartiles. -gluc: -glucuronide.

In the plasma, berberine and all phase I metabolites were below the limit of quantification (LOQ = 0.1 ng/mL), with the exception of thalifendine (0.66 ± 0.4 ng/mL). Phase II metabolites were the most abundant forms of berberine in the plasma, particularly thalifendine glucuronide (Figure 1). Phase II metabolites have been shown to be the major metabolites detected in the blood of rats and chickens [18,52]. All phase II metabolites were a result of glucuronidation, and no phase II metabolites resulting from sulfation were detected. This may be due to the lack of or the reduced capacity of sulfate conjugation in pigs [53].

**Figure 2:**
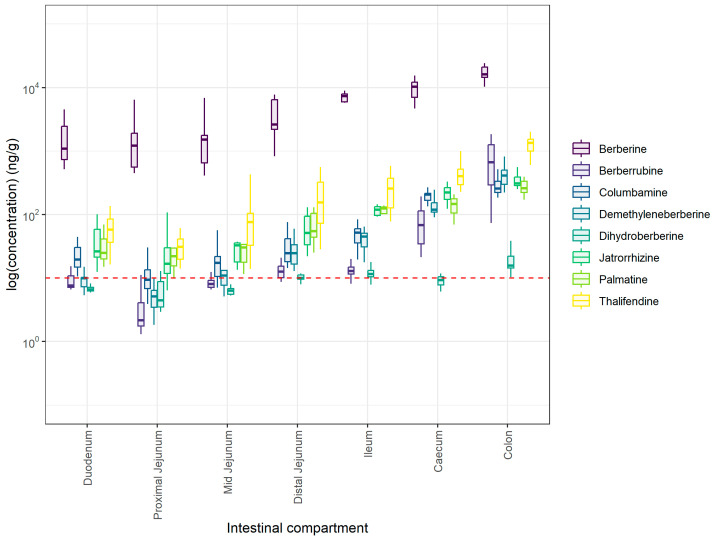
Berberine and phase I metabolite concentrations in small and large intestinal contents of 6-week-old piglets fed 30 mg BBR/kg feed for 2 weeks (n = 12). Red dotted line represents the limit of quantification (LOQ) established at 5 ng/g during method validation experiments, while whiskers represent the range within 1.5 × IQR from the quartiles. LOQ of dihydroberberine was established at 10 ng/g.

Berberine and metabolites were also quantified in the intestinal contents of all intestinal segments from the duodenum to the colon (Figure 2). Berberine was detected in all intestinal compartments, as well as its phase I metabolites, with increasing concentrations from the proximal to the distal part of the gastrointestinal tract (GIT) (Supplementary Table S2), reaching a peak in the cecum and the colon (Figure 2). More particularly, berberine was significantly increased in the distal jejunum compared to the more proximal segments of the small intestine. Concentrations in the cecum but not in the ileum were higher compared to the distal jejunum (*p* = 0.0189), and similar to the colon (*p* = 0.3790). The same trend observed for berberine was also observed for phase I metabolites, with increased concentrations along the intestinal compartments. Thalifendine was the most abundant phase I metabolite across all the compartments (*p* < 0.001) (Figure 2; Supplementary Table S2). Berberrubine concentrations were below the LOQ (5 ng/g) in the jejunum, suggesting negligible concentrations in this region. The dihydroberberine concentration was above LOQ (10 ng/g) only in the distal jejunum, ileum, and colon (Figure 2).

Berberine was previously found to be extensively demethylated to thalifendine and berberrubine, as well as converted to demethyleneberberine to a smaller extent, by cecal and colonic microbiota in vitro, and not by jejunal and ileal flora originating from broiler chickens [18]. While concentrations of berberine were not significantly different between cecum and colon, thalifendine, berberrubine, demethyleneberberine, and palmatine levels were more elevated in the colon compared to the cecum (Supplementary Table S2), which could imply a more extensive metabolism, including demethylation, in the colon of pigs; therefore, species differences might be in effect.

Phase II metabolites were also quantified in the intestinal contents (Supplementary Table S3). Thalifendine–glucuronide, berberrubine–glucuronide, columbamine–glucuronide, and jatrorrhizine–glucuronide appeared to have relatively stable concentrations in the small intestine but significantly lower concentrations in the cecum (*p* < 0.0001), and they were not detected in the colon. Demethyleneberberine–glucuronide 01, 02, and 05 were quantified in the small intestine, but not in the large intestine. Demethyleneberberine-glucuronide 03, 04, and 06 were significantly quantified in the distal jejunum and borderline in the duodenum. However, none of these forms were detected in the cecum or colon (Supplementary Figures S2–S4). Regarding the sulfate conjugates, berberrubine sulfate and jatrorrhizine sulfate were quantified solely in the distal jejunum, while concentrations were below the LOQ in the remainder of the compartments and not detected in the large intestine, hence not significantly present in the intestine. The absence or lower concentrations of phase II metabolites in the large intestine compared to the small intestine was also seen in chickens fed a berberine-supplemented diet and could be due to higher bacterial glucuronidase and sulfatase activity that deconjugates phase II metabolites originating from the liver via entero-hepatic circulation back to their associated phase I metabolites [18]. This potential higher glucuronidase activity in the large intestine could also explain why we see elevated levels of phase I metabolites in the cecum and/or colon compared to the small intestine (Supplementary Table S2). More specifically, thalifendine–glucuronide was predominant in plasma, suggesting it is a major phase II metabolite produced in the liver of piglets. Its presence in the intestine following enterohepatic circulation and subsequent deconjugation may explain the consistently high concentrations of thalifendine observed throughout all intestinal segments. Berberine metabolites can be products of the host and/or the gut microbiota metabolism. Particularly, Feng et al., 2015 showed that gut bacteria transform berberine to dihydroberberine [29], while Dehau et al., 2023 showed that thalifendine is derived from both host and gut bacteria metabolism, whereas berberrubine seems to be mainly produced by gut microbiota [18].

The investigation of berberine metabolism after administration is of interest because metabolites are believed to play an important role in berberine’s biological effects [19]. Berberrubine, but not berberine, has been shown to increase SCFA production in vitro in cecal microbial cultures [18].

Furthermore, some of berberine’s metabolites may have antibacterial actions. Studies showed that berberine and its phase I metabolites, palmatine and jatrorrhizine, have inhibitory effects against pathogens like *E. coli* or *Salmonella* Typhi in vitro. It is worth noting that the effective concentrations reported in vitro (100–800 µg/mL) are relatively high and that higher dietary levels than the one used in this study would be necessary to achieve comparable antibacterial effects in vivo [54,55]. Antibacterial properties can be of importance in the swine industry since ETEC infection is a major cause of PWD in piglets.

Berberine metabolites can also lower oxidative stress. Increased levels of malondialdehyde in the blood after weaning suggest that weaning causes oxidative stress in piglets, which can ultimately lead to gut barrier injury and impaired nutrient absorption [56,57]. During oxidative stress, there is an excess of reactive oxygen species (ROS) that cannot be scavenged in time. This presents a problem, as ROS are unstable and contain hydroxyl radicals (•OH), which are some of the most reactive products and can cause damage to lipids and DNA [58,59]. Jang et al., 2009 indicated that berberrubine had a stronger •OH scavenging activity than berberine in vitro, although at much higher concentrations than those found in our study, by suppressing its generation rather than capturing it [60].

In addition, berberine metabolites have been shown to have anti-inflammatory properties. Yan et al., 2017 showed that palmatine could reduce LPS-induced inflammation in goat endometrial epithelial cells (gEECs) by inhibiting the production of pro-inflammatory cytokines TNF-α, IL-6, and IL-1β in a dose-dependent manner [61]. Berberrubine and dihydroberberine downregulated pro-inflammatory cytokines and upregulated tight junction proteins in a mouse colitis model [62,63]. Interestingly, the metabolism of berberine was studied in vitro in the intestinal microbiota isolated from piglets with diarrhea and from control piglets, and nitroreductase activity was higher in diarrheal intestinal microbiota compared to the control [64]. This enzyme is responsible for the conversion of berberine into dihydroberberine. Accordingly, berberine was more extensively metabolized in the diarrheal intestinal flora. This improved metabolism may enhance berberine’s effects via the production of bioactive metabolites. These anti-inflammatory properties of berberine metabolites could be useful during weaning and/or ETEC infection, which are known to increase pro-inflammatory cytokines that are detrimental to gut health [65,66].

## 4. Conclusions

In this study, we investigated the effect of a low dose of berberine on the intestinal health of weaned piglets after 2 weeks of supplementation in the feed. At a low dose (0.03 g/kg), berberine did not significantly affect small intestinal morphology, SCFA production, or the gut microbiota. Thalifendine and glucuronide forms of berberine phase I metabolites were the main metabolites of berberine in plasma, exceeding the concentration of berberine itself. Berberine and phase I metabolites concentrations increased from the duodenum toward the colon. Relatively higher concentrations of berberine in feed may be needed to observe beneficial effects on gut health, as well as more significant levels of bioactive berberine-derived metabolites reaching the systemic circulation. More studies, including studies under challenged conditions, are needed to optimize the dose for commercial use and to investigate exactly how berberine, along with its metabolites, can aid in counteracting the adverse effects of early weaning.

## Figures and Tables

**Table 1:** Composition of the basal diet.

Ingredients	%
Barley	26.677
Wheat	25
Soybeans	13.083
Corn	10
Premix weaner	9
Soybean meal	6
Beet molasses	3
Potato protein	2
Wheat gluten	1.571
Monocalciumphosphate	0.923
Limestone	0.747
L-Lysine HCL	0.604
Salt	0.492
L-threonine	0.259
DL-methionine	0.243
L-Valine	0.142
L-Tryptophan	0.079
Ronozyme (500 FYT)	0.05
Ronozyme (1000 FYT)	0.05
Leucine valine 90/10	0.049
Isoleucine valine 50/50	0.033

**Table 2:** Effect of berberine on small intestinal morphology and body weight.

Parameters	Control	BBR	*p* Value
Duodenum			
Villus height (μm)	408.92 ± 43.99	410.43 ± 89.79	0.347
Crypt depth (μm)	368 ± 122.5	437.41 ± 65.59	0.0045
Villus/Crypt ratio	1.19 ± 0.32	0.98 ± 0.37	0.088
Proximal Jejunum			
Villus height (μm)	401.79 ± 97.41	320.31 ± 94.17	0.061
Crypt depth (μm)	300.22 ± 86.68	342.93 ± 93.25	0.283
Villus/Crypt ratio	1.44 ± 0.49	1.02 ± 0.46	0.0484
Middle Jejunum			
Villus height (μm)	369.57 ± 61.32	330.73 ± 87.75	0.464
Crypt depth (μm)	281.49 ± 76.31	330.02 ± 83.75	0.183
Villus/Crypt ratio	1.43 ± 0.49	1.12 ± 0.56	0.188
Distal Jejunum			
Villus height (μm)	291.9 ± 53.83	305.34 ± 96.93	0.689
Crypt depth (μm)	256.52 ± 89.74	288.4 ± 72.17	0.357
Villus/Crypt ratio	1.27 ± 0.46	1.18 ± 0.62	0.727
Ileum			
Villus height (μm)	295.34 ± 56.34	299.79 ± 69.03	0.873
Crypt depth (μm)	224.9 ± 74.70	280.83 ± 78.92	0.043
Villus/Crypt ratio	1.45 ± 0.51	1.17 ± 0.46	0.128
Body weight (6 weeks)	11.2 kg ± 1.1	11.3 kg ± 1.4	0.803

Data represent the mean ± standard deviation. Analysis was based on 7 measurements per section (n = 12) for villus height and crypt depth.

**Table 3:** Alpha diversity index in mid jejunum (n = 12), cecum (n = 12), and colon (n = 12) contents.

	Chao1 Index		
**Intestinal segment**	**BBR**	**Control**	***p* Value**
Mid Jejunum	115.72 ± 102.39	122.73 ± 107.41	0.87
Cecum	381.8 ± 92.3	331.6 ± 70.7	0.16
Colon	573.84 ± 119.9	526.79 ± 92.49	0.27

**Table 4:** SCFA quantification in cecal (n = 12) and colonic (n = 12) contents.

SCFA (µmol/g)	Cecum		*p* Value	Colon		*p* Value
	BBR	Control		BBR	Control	
Acetic Acid	71.28	69.46	0.740	78.91	74.32	0.299
Propionic Acid	41.75	42.23	0.918	33.84	32.48	0.659
Butyric Acid	15.43	15.61	0.954	16.51	15.93	0.776
Valeric Acid	2.02	3.12	0.272	3.09	3.91	0.138
Caproic Acid	0.14	0.20	0.146	0.27	0.28	0.916
Isobutyric Acid	0.28	0.37	0.065	1.36	1.48	0.59
Isovaleric Acid	0.31	0.38	0.270	1.72	1.91	0.557
Isocaproic Acid	0.13	0.10	0.291	0.10	0.09	0.206
Total bCFAs	0.72	0.85	0.291	3.18	3.48	0.562
Total SCFAs	131.34	131.47	0.992	135.80	130.40	0.512

bCFAs: branched-chain fatty acids.

## Data Availability

The sequencing data are available on NCBI SRA under the BioProject PRJNA1268895. All other original contributions presented in this study are included in the article, and the raw data supporting the conclusions of this article will be made available by the authors upon request.

## Supplementary Materials

The following supporting information can be downloaded at: https://www.mdpi.com/article/10.3390/ani15162450/s1, Supplementary Materials S1: Figure S1: Influence of sow on piglet microbiota; Figure S2: Phase II glucuronide conjugated metabolite concentrations in small and large intestinal content of 6-week-old piglets fed 30 mg BBR/kg feed for 2 weeks (n = 12); Figure S3: Phase II glucuronide conjugated metabolite concentrations in small and large intestinal content of 6-week-old piglets fed 30 mg BBR/kg feed for 2 week (n = 12); Figure S4: Phase II sulfate conjugated metabolite concentrations in small and large intestinal content of 6-week-old piglets fed 30 mg BBR/kg feed for 2 weeks (n = 12); Table S1: Trial set-up. Piglet distribution between two treatment groups with weights at the start (4 weeks old) and end (6 weeks old) of the trial; Table S2: BBR phase I metabolites; Table S3: BBR phase II metabolites; Supplementary Materials S2: method validation [30,31,32,67,68].

## Abbreviations

The following abbreviations are used in this manuscript:

SCFAs	Short-chain fatty acids.
PWD	Post-weaning diarrhea.
BBR	Berberine.
LOQ	Limit of quantification.
UPLC-MS/MS	Ultra performance liquid chromatography-tandem mass spectrometry.
ROS	Reactive oxygen species.
CTR	Control.
ZnO	Zinc oxide.
ETEC	Enterotoxigenic *Escherichia coli.*
CTAB	Cetyltrimethylammonium bromide.
bCFA	Branched-Chain Fatty Acids.
GIT	Gastrointestinal tract.
•OHs	Hydroxyl radicals.
gEECs	Goat endometrial epithelial cells.
