# Supplementary material for: A Low Dose of Berberine Is Metabolized in Weaned Piglets Without Major Changes to Gut Morphology or Gut Microbiota
Source: Animals (Basel). 2025 Aug 21;15(16):2450. doi: 10.3390/ani15162450 (PMC12383199; doi:10.3390/ani15162450)
Supplement: Supplementary file 1 [file animals-15-02450-s001.zip › Supplementary Materials_S1.pdf]

Supplementary Materials S1

Table S1. Trial set-up. Piglet distribution between two treatment groups with weights at the start (4 weeks old) and end (6 weeks old) of the trial.

SowID	PigID	Treatment	Pen	Weight at 4 weeks (kg)	Weight at 6 weeks (kg)
420-22	892	Control	CTR1	7.6	9.3
420-22	895	Control	CTR2	7.6	10.6
420-22	893	BBR	BBR1	9.8	11.4
420-22	897	BBR	BBR2	7.7	9.9
407-22	806	Control	CTR1	8.8	11.4
407-22	817	Control	CTR2	9.0	12.6
407-22	812	BBR	BBR1	9.3	11.5
407-22	819	BBR	BBR2	9.2	10.6
394-22	744	Control	CTR3	8.5	10.8
394-22	748	Control	CTR4	9	12.8
394-22	746	BBR	BBR3	10.0	14.8
394-22	750	BBR	BBR4	10.3	12.9
263-18	916	Control	CTR3	8.3	9.5
263-18	919	Control	CTR4	7.7	11.4
263-18	918	BBR	BBR3	8.3	10.6
263-18	920	BBR	BBR4	8.2	9.9
364-21	793	Control	CTR5	8.8	11.3
364-21	795	Control	CTR6	8.3	10.4
364-21	794	BBR	BBR5	8.7	11.6
364-21	796	BBR	BBR6	7.4	9.6
359-21	760	Control	CTR5	9.2	12.6
359-21	765	Control	CTR6	8.4	11.8
359-21	762	BBR	BBR5	8.5	11.7
359-21	767	BBR	BBR6	8.9	11.3

Feed sample preparation procedure

About 50 g of pelleted feed was grinded in a Moulinette mixer. A 1.25 g subsample of the grinded feed was weighed accurately in a plastic 50 mL centrifuge tube. Subsequently, 50 µL of an internal standard solution containing 250 µg/mL of berberine-d6 in water were added, followed by brief vortex mixing. Thereafter, a 50 mL volume of a 1 % (v/v) formic acid solution in methanol was added as the extraction solvent. Extraction of the sample was obtained by successively incubation for 1 h in an ultrasonic bath, shaking during 1 h on a rotary shaker at 80 rpm, incubation for 1 h in an ultrasonic bath, and shaking during 1 h on a rotary shaker at 80 rpm. After the final incubation step, the sample was centrifuged (4000 rpm, 10 min., 4 °C). Thereafter, the supernatant was diluted a factor 1/100 in a 15 mL glass tube with water as follows: 100 µL sample extract + 9.9 mL Milli-Q water, followed by vortex mixing (15 s on the vortex instrument at 2500 rpm). The 1/100 diluted sample extract was further diluted a factor 1/10 directly in a conical glass autosampler vial as follows: 100 µL sample extract (diluted 1/100) + 900 µL 0.1 % (v/v) formic acid in water, followed by brief vortex mixing. As such, at the 30 mg berberine/kg feed target level, this corresponds to a final nominal concentration of

0.75 ng/mL berberine in the injected sample extract. A 5 µL sample aliquot was injected onto the LC-MS/MS apparatus.

Calibrator and all other spiked samples (quality control samples) were prepared as follows: 1.25 g blank feed (normal pelleted pig feed containing no berberine grinded in a Moulinette mixer) + a volume ranging from 62.5 to 150 µL of a 300 µg/mL solution of berberine in HPLC methanol + 50 µL of an internal standard solution containing 250 µg/mL of berberine-d6 in water. The calibration curve was in the 50 – 120 % range of the target level, and included the levels 50, 60, 70, 80, 89.6, 100, 109.6, and 120 % of the target level. Quantification was based on the ratio of the berberine peak area compared to the peak area of the internal standard berberine-d6.

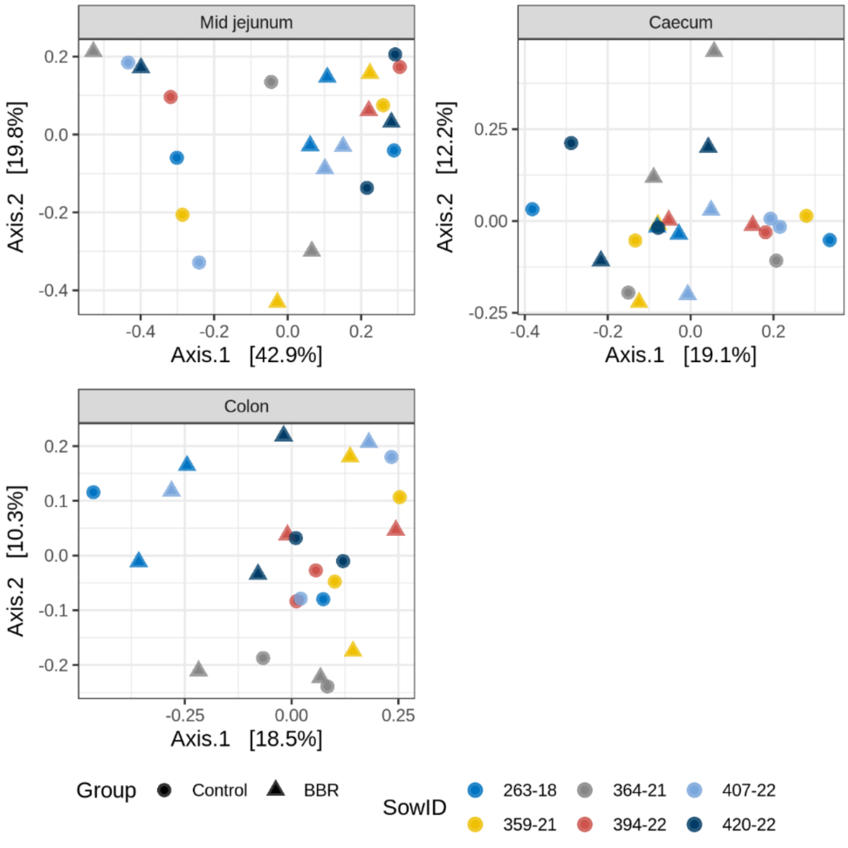

Figure S1. Influence of sow on piglet microbiota. A significant effect of the sow on the colonic microbiota of the piglets was observed, explaining 30% of the variation between the samples (p=0.001). This effect is less pronounced in the mid jejunum and cecum where there was no influence of the sowID on the microbiota observed, and the piglets from the same sow are more widely dispersed. Each dot and triangle represent the microbiota of a piglet.

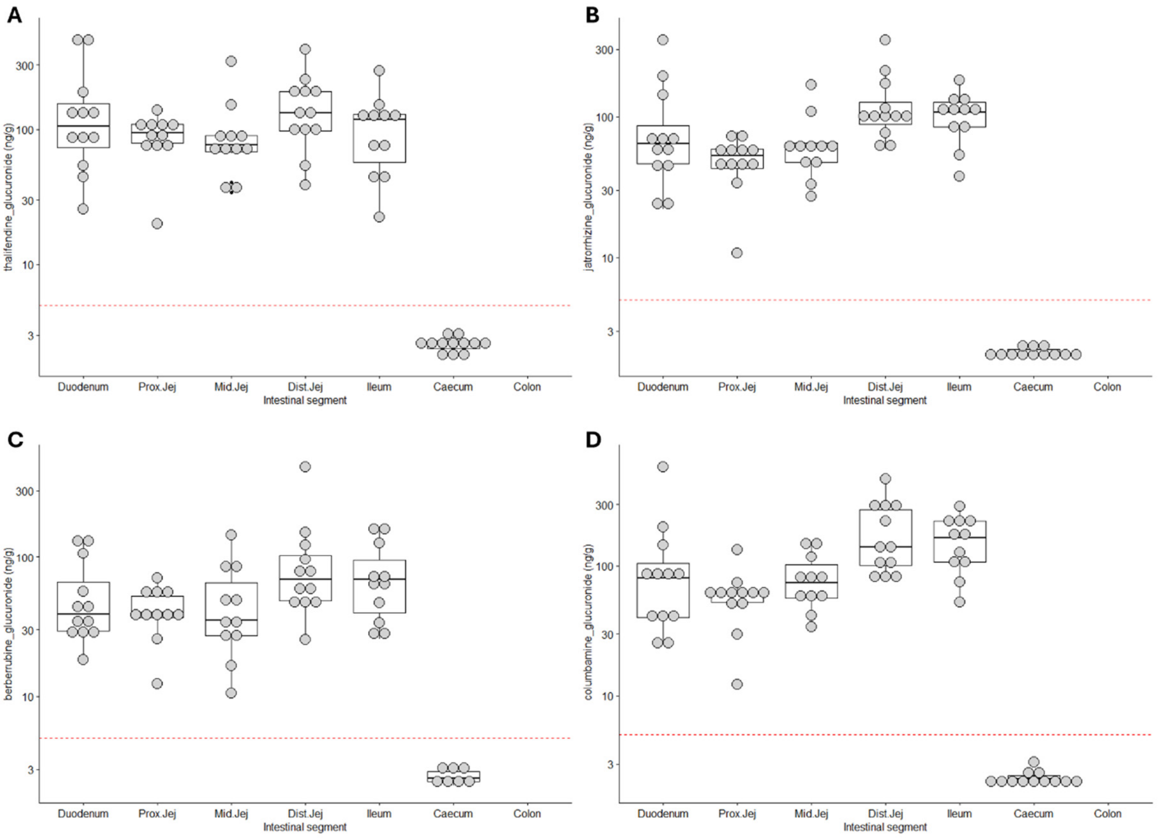

Figure S2. Phase II glucuronide conjugated metabolite concentrations in small and large intestinal content of 6-week-old piglets fed 30 mg BBR/kg feed for 2 weeks (n=12). Red dotted line represents the Limit of Quantification (LOQ) established at 5 ng/g during method validation experiments while whiskers represent the range within 1.5 × IQR from the quartiles. A)Thalifendine glucuronide. B) Berberrubine glucuronide. C) Columbamine glucuronide. D) Jatrorrhizine-glucuronide

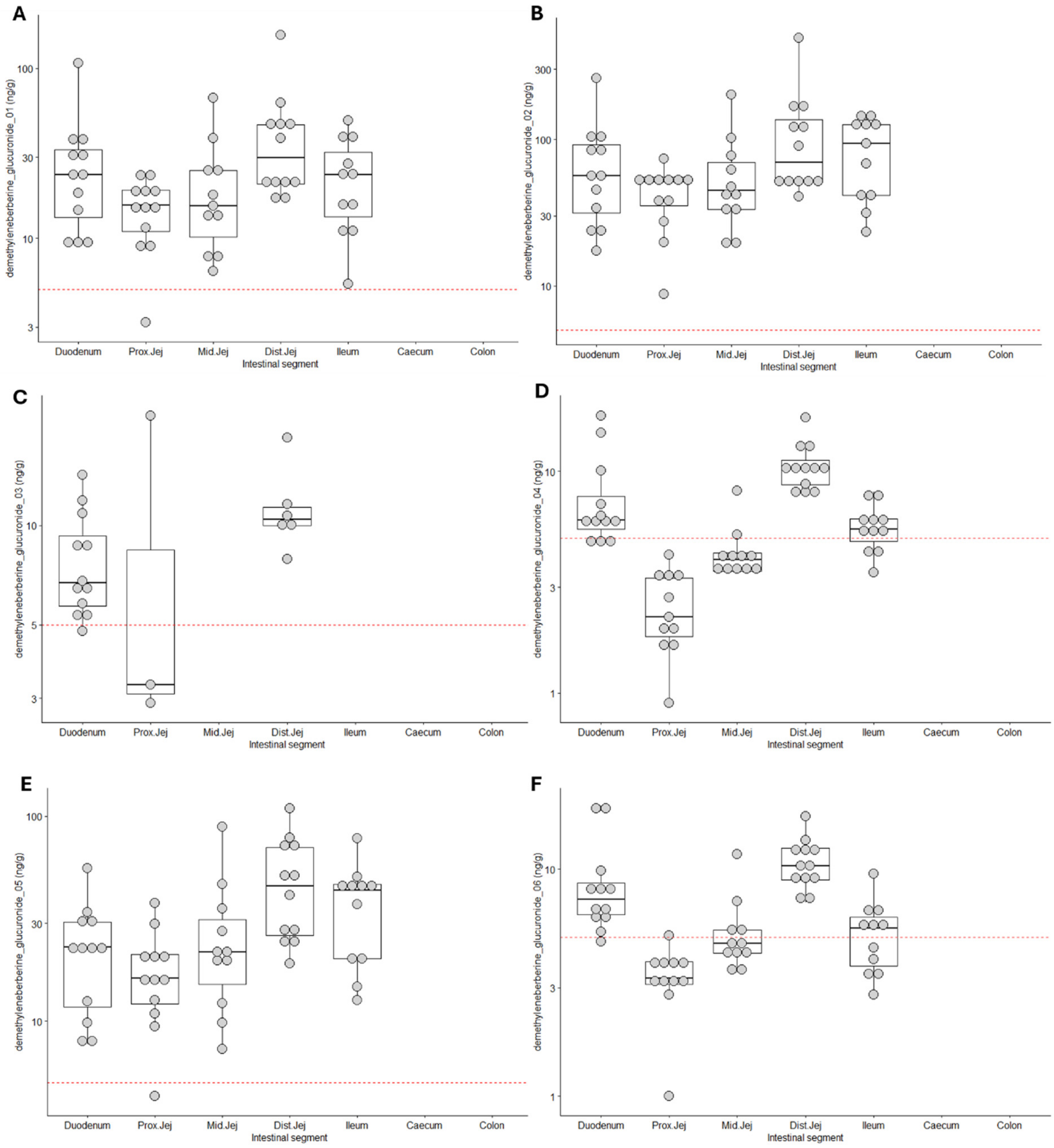

Figure S3. Phase II glucuronide conjugated metabolite concentrations in small and large intestinal content of 6-week-old piglets fed 30 mg BBR/kg feed for 2 week (n=12). Red dotted line represents the Limit of Quantification (LOQ) established at 5 ng/g during method validation experiments while whiskers represent the range within 1.5 × IQR from the quartiles. A) Demethyleneberberine-glucuronide_01. B) Demethyleneberberine-glucuronide_02. C) Demethyleneberberine-glucuronide_03. D) Demethyleneberberine-glucuronide_04. E) Demethyleneberberine-glucuronide_05. F) Demethyleneberberine-glucuronide_06.

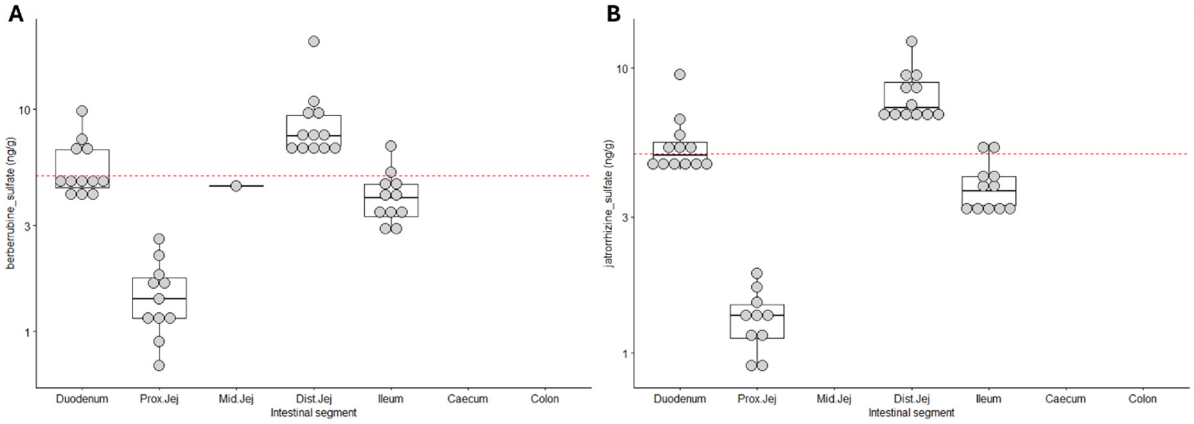

Figure S4. Phase II sulfate conjugated metabolite concentrations in small and large intestinal content of 6-week-old piglets fed 30 mg BBR/kg feed for 2 weeks (n=12). Red dotted line represents the Limit of Quantification (LOQ) established at 5 ng/g during method validation experiments while whiskers represent the range within 1.5 × IQR from the quartiles.. A) Berberrubine-sulfate. B) Jatrorrhizine-sulfate.
