# Supplementary material for: A Low Dose of Berberine Is Metabolized in Weaned Piglets Without Major Changes to Gut Morphology or Gut Microbiota
Source: Animals (Basel). 2025 Aug 21;15(16):2450. doi: 10.3390/ani15162450 (PMC12383199; doi:10.3390/ani15162450)
Supplement: Supplementary file 1 [file animals-15-02450-s001.zip › Supplementary materials_S2_method validation.pdf]

Supplementary Methods

Supplementary Methods S2 – Validation results of the quantification of berberine and berberine-derived metabolites in plasma and intestinal contents of pig by UPLC-MS/MS. The analytical method was evaluated in terms of the validation criteria described by the CVMP (EMEA/CVMP/VICH/463202/2009) and CHMP (EMA/CHMP/ICH/172948/2019) guidelines.

PLASMA SAMPLES

Quantification of all analytes in plasma of pig was based on the internal standard berberine-d6, except for dihydroberberine where tetrahydropalmatine was used as the internal standard. Thalifendine was quantified using the berberrubine calibration curve and phase II metabolites were quantified relative to the berberine calibration curve, since for these compounds no analytical reference standards were available.

Linearity

Matrix-matched calibration curves were prepared on three different analysis days in the concentration range 0.1 – 100 ng/ml, including the levels 0.1, 0.25, 0.5, 1, 2.5, 5, 10, 25, 50, and 100 ng/ml. Calibration curves were best described by a linear calibration model y= ax + b, using a $1/x^2$ fit weighting, resulting as such in the lowest goodness-of-fit coefficient (GoF) %.

As can be seen, the correlation coefficient (r) was higher than the 0.99 criterion, while the GoF (%) was below the 20% criterion for all components.

Linearity evaluation for berberine and phase I metabolites in pig plasma, concentration range: 0.1 – 100 ng/ml.

**Compound**	**Evaluation occasion**	**a**	**b**	**r**	**GoF (%)**
**Berberine**	Day 1	0.2762	0.000584	0.9983	5.2
	Day 2	0.2752	0.001665	0.9985	4.9
	Day 3	0.2753	-0.003189	0.9989	4.1
**Demethyleneberberine**	Day 1	0.1703	0.003559	0.9990	3.9
	Day 2	0.1731	0.002501	0.9946	6.5
	Day 3	0.1789	-0.000879	0.9991	3.8
**Columbamine**	Day 1	0.1353	-0.000649	0.9981	5.5
	Day 2	0.1402	-0.001385	0.9987	4.6
	Day 3	0.1398	-0.001778	0.9985	4.9

**Jatrorrhizine**	Day 1	0.1896	-0.001896	0.9988	4.3
	Day 2	0.1938	0.001365	0.9991	3.7
	Day 3	0.1941	-0.001069	0.9992	3.5
**Berberrubine**	Day 1	0.5590	-0.009181	0.9981	5.4
	Day 2	0.5915	-0.007591	0.9983	5.1
	Day 3	0.5877	-0.012050	0.9984	5.0
**Dihydroberberine**	Day 1	0.05900	-0.000208	0.9973	10.5
	Day 2	0.05972	-0.000500	0.9982	5.3
	Day 3	0.05841	-0.000429	0.9965	7.4
**Palmatine**	Day 1	0.1967	0.006847	0.9962	7.7
	Day 2	0.1971	0.009313	0.9982	5.3
	Day 3	0.1969	0.005021	0.9985	4.8

Accuracy and precision, and limit of quantification

At each level evaluated for accuracy and precision, i.e. 0.1, 1, 10, and 100 ng/ml, 6 independent measurements were performed at each of three analysis days.

Tolerances were as follows for within-day accuracy: -20% to +10% at 10 and 100 ng/ml levels, and -50 to +20% at 0.1 and 1 ng/ml levels (Heitzman, 1994; EU legislation, 1991, 2003, 2004). Maximum tolerance for within-day precision (relative standard deviation, RSD) was concentration dependent: 10%, 15%, 25%, and 30% at the 100, 10, 1, and 0.1 ng/ml level, respectively (VICH GL49, 2016). Criteria were met for within-day accuracy and precision for all compounds at each concentration level.

Related to between-day accuracy, the above-mentioned criteria were met for all components at each level tested, and between-day precision criteria were also met, as calculated according to the Horwitz equation: 22.6%, 32.0%, 45.3%, and 64.0% at the 100, 10, 1, and 0.1 ng/ml level, respectively (Heitzman, 1994; EU legislation, 2003, 2004).

Based on the above, the limit of quantification (LOQ) was established at 0.1 ng/ml in pig plasma for all compounds included.

Within-day and between-day accuracy and precision evaluation for berberine and phase I metabolites in pig plasma at 0.1, 1, 10, and 100 ng/ml levels.

Compound	Evaluation occasion	Level 1: 0.1 ng/ml	Level 2: 1 ng/ml	Level 3: 10 ng/ml	Level 4: 100 ng/ml
**Berberine**	**Day 1**	0.10 ± 0.003 (acc +4.2%) RSD 2.7%	0.95 ± 0.04 (acc -5.1%) RSD 4.2%	10.2 ± 0.2 (acc +2.2%) RSD 2.4%	100.3 ± 2.1 (acc +0.3%) RSD 2.1%
	**Day 2**	0.10 ± 0.006 (acc +3.5%) RSD 6.0%	0.92 ± 0.04 (acc -8.3%) RSD 4.0%	10.4 ± 0.2 (acc +4.5%) RSD 2.0%	101.3 ± 3.3 (acc +1.3%) RSD 3.2%
	**Day 3**	0.11 ± 0.003 (acc +12.5%) RSD 3.0%	0.94 ± 0.03 (acc -5.8%) RSD 2.7%	10.7 ± 0.2 (acc +7.1%) RSD 1.8%	105.0 ± 2.3 (acc +5.0%) RSD 2.2%
	**Between-day**	0.11 ± 0.006 (acc +6.7%) RSD 5.5%	0.94 ± 0.04 (acc -6.4%) RSD 3.8%	10.5 ± 0.3 (acc +4.6%) RSD 2.8%	102.2 ± 3.2 (acc +2.2%) RSD 3.1%
**Demethyleneberberine**	**Day 1**	0.10 ± 0.017 (acc -0.2%) RSD 16.6%	1.09 ± 0.15 (acc +8.8%) RSD 13.5%	11.0 ± 0.5 (acc +9.6%) RSD 4.4%	99.3 ± 2.5 (acc -0.7%) RSD 2.5%
	**Day 2**	0.093 ± 0.012 (acc -7.0%) RSD 13.0%	1.06 ± 0.08 (acc +6.4%) RSD 7.5%	10.5 ± 0.5 (acc +4.8%) RSD 4.3%	95.1 ± 2.3 (acc -4.9%) RSD 2.4%
	**Day 3**	0.097 ± 0.005 (acc -3.3%) RSD 5.6%	0.95 ± 0.03 (acc -5.0%) RSD 2.8%	10.3 ± 0.1 (acc +2.8%) RSD 1.5%	102.9 ± 2.7 (acc +2.9%) RSD 2.7%
	**Between-day**	0.097 ± 0.012 (acc -3.5%) RSD 12.3%	1.03 ± 0.11 (acc +3.4%) RSD 10.7%	10.6 ± 0.5 (acc +5.7%) RSD 4.5%	99.1 ± 4.0 (acc -0.9%) RSD 4.1%
**Columbamine**	**Day 1**	0.098 ± 0.013 (acc -1.8%) RSD 13.6%	0.97 ± 0.06 (acc -3.0%) RSD 6.0%	10.6 ± 0.3 (acc +5.6%) RSD 2.8%	104.6 ± 4.2 (acc +4.6%) RSD 4.0%
	**Day 2**	0.10 ± 0.010 (acc +2.7%) RSD 10.0%	0.94 ± 0.03 (acc -5.6%) RSD 3.6%	10.1 ± 0.2 (acc +1.2%) RSD 2.0%	99.5 ± 2.4 (acc -0.5%) RSD 2.4%
	**Day 3**	0.10 ± 0.009 (acc +3.0%) RSD 8.8%	0.94 ± 0.02 (acc -5.5%) RSD 2.1%	10.4 ± 0.2 (acc +3.8%) RSD 1.6%	103.6 ± 2.1 (acc +3.6%) RSD 2.1%
	**Between-day**	0.10 ± 0.011 (acc +1.3%) RSD 10.5%	0.95 ± 0.04 (acc -4.7%) RSD 4.2%	10.3 ± 0.3 (acc +3.5%) RSD 2.7%	102.6 ± 3.7 (acc +2.6%) RSD 3.6%
**Jatrorrhizine**	**Day 1**	0.11 ± 0.008 (acc +10.0%) RSD 7.4%	1.03 ± 0.09 (acc +2.9%) RSD 8.6%	10.7 ± 0.3 (acc +6.7%) RSD 3.1%	102.0 ± 2.7 (acc +2.0%) RSD 2.6 %
	**Day 2**	0.090 ± 0.006 (acc -10.3%) RSD 6.5%	1.00 ± 0.07 (acc -0.3%) RSD 6.7%	10.3 ± 0.3 (acc +3.3%) RSD 2.6%	99.3 ± 2.8 (acc -0.7%) RSD 2.8%
	**Day 3**	0.10 ± 0.008 (acc +4.3%) RSD 7.2%	0.95 ± 0.03 (acc -5.2%) RSD 3.2%	10.4 ± 0.2 (acc +4.2%) RSD 1.6%	101.8 ± 2.1 (acc +1.8%) RSD 2.1%
	**Between-day**	0.10 ± 0.011 (acc +1.3%) RSD 11.0%	0.99 ± 0.07 (acc -0.9%) RSD 7.2%	10.5 ± 0.3 (acc +4.7%) RSD 2.7%	101.0 ± 2.7 (acc +1.0%) RSD 2.7%
**Berberrubine**	**Day 1**	0.11 ± 0.009 (acc +5.8%) RSD 8.3%	0.93 ± 0.04 (acc -7.0%) RSD 4.3%	10.2 ± 0.3 (acc +1.7%) RSD 2.5%	94.8 ± 2.4 (acc -5.2%) RSD 2.6%

	**Day 2**	0.097 ± 0.006 (acc -3.5%)<br>RSD 5.9%	0.83 ± 0.02 (acc -17.4%)<br>RSD 2.1%	9.5 ± 0.5 (acc -5.0%)<br>RSD 4.8%	91.6 ± 1.8 (acc -8.4%)<br>RSD 2.0%
	**Day 3**	0.10 ± 0.003 (acc -0.3%)<br>RSD 3.3%	0.89 ± 0.02 (acc -11.3%)<br>RSD 2.0%	10.2 ± 0.3 (acc +2.1%)<br>RSD 2.8%	97.0 ± 1.9 (acc -3.0%)<br>RSD 1.9%
	**Between-day**	0.10 ± 0.007 (acc +0.7%)<br>RSD 7.1%	0.88 ± 0.05 (acc -11.9%)<br>RSD 5.8%	10.0 ± 0.5 (acc -0.4%)<br>RSD 4.6%	94.4 ± 3.0 (acc -5.6%)<br>RSD 3.1%
**Dihydroberberine**	**Day 1**	0.094 ± 0.009 (acc -6.2%)<br>RSD 9.5%	0.94 ± 0.09 (acc -5.6%)<br>RSD 9.6%	10.4 ± 0.6 (acc +3.8%)<br>RSD 5.5%	100.9 ± 1.6 (acc +0.9%)<br>RSD 1.6%
	**Day 2**	0.098 ± 0.007 (acc -1.7%)<br>RSD 7.2%	0.95 ± 0.07 (acc -5.1%)<br>RSD 7.3%	9.5 ± 0.5 (acc -5.0%)<br>RSD 5.1%	99.2 ± 4.2 (acc -0.8%)<br>RSD 4.2%
	**Day 3**	0.091 ± 0.010 (acc -8.8%)<br>RSD 10.5%	0.95 ± 0.02 (acc -4.5%)<br>RSD 2.0%	9.6 ± 0.2 (acc -4.4%)<br>RSD 2.5%	96.1 ± 2.8 (acc -3.9%)<br>RSD 2.9%
	**Between-day**	0.094 ± 0.009 (acc -5.6%)<br>RSD 9.1%	0.95 ± 0.06 (acc -5.1%)<br>RSD 6.6%	9.8 ± 0.6 (acc -1.9%)<br>RSD 6.0%	98.7 ± 3.5 (acc -1.3%)<br>RSD 3.6%
**Palmatine**	**Day 1**	0.11 ± 0.01 (acc +12.0%)<br>RSD 12.5%	0.89 ± 0.04 (acc -10.6%)<br>RSD 4.0%	10.1 ± 0.2 (acc +0.6%)<br>RSD 2.1%	101.8 ± 2.7 (acc +1.8%)<br>RSD 2.6%
	**Day 2**	0.091 ± 0.011 (acc -9.2%)<br>RSD 12.6%	0.87 ± 0.03 (acc -12.6%)<br>RSD 3.5%	9.7 ± 0.4 (acc -3.0%)<br>RSD 4.6%	101.3 ± 3.4 (acc +1.3%)<br>RSD 3.4%
	**Day 3**	0.099 ± 0.007 (acc -1.4%)<br>RSD 6.8%	0.91 ± 0.02 (acc -9.0%)<br>RSD 2.5%	10.1 ± 0.3 (acc +0.9%)<br>RSD 2.6%	101.2 ± 2.0 (acc +1.2%)<br>RSD 1.9%
	**Between-day**	0.10 ± 0.014 (acc +0.6%)<br>RSD 14.0%	0.89 ± 0.03 (acc -10.7%)<br>RSD 3.6%	9.9 ± 0.4 (acc -0.5%)<br>RSD 3.6%	101.4 ± 2.6 (acc +1.4 %)<br>RSD 2.6%

Mean values ± SD (n=6) are given, as well as accuracy (acc, % deviation from nominal value), and precision (as % RSD). One outlier value was excluded for calculations: for palmatine at the 0.1 ng/ml level, repl-05 value at day 3, measured as 3.9 ng/ml.

Specificity and carry-over

The specificity was demonstrated by the analysis of blank pig plasma, i.e. without the addition of analytes nor IS compounds. No peaks of berberine or related metabolites were detected in these samples. Also peaks of berberine-d6 or tetrahydropalmatine were absent (results not shown). Carry-over was evaluated by the injection of a solvent after the injection of the highest 100 ng/ml calibrator sample. No peaks were detected in this solvent injection, so carry-over was virtually absent at the concentration levels involved (results not shown).

Stability in processed sample extract

At each level evaluated, i.e. 0.1, 1, 10 and 100 ng/ml, 3 replicates of prepared sample extracts were re-injected after a 24 h storage time at 10 °C (in the autosampler compartment), and quantified using a freshly prepared matrix-matched calibration curve.

The criteria for accuracy and precision were met (see section on accuracy and precision for criteria) for all components, including dihydroberberine, indicating that the addition of ascorbic acid during sample preparation effectively succeeded in stabilizing this unstable compound.

Stability of berberine and phase I metabolites in processed pig plasma sample extracts at the 0.1, 1, 10, and 100 ng/ml levels, stored at 10 °C for 24 h.

Compound	Level 1: 0.1 ng/ml	Level 2: 1 ng/ml	Level 3: 10 ng/ml	Level 4: 100 ng/ml
**Berberine**	0.10 ± 0.007 (acc +0.3%)<br>RSD 7.0%	1.00 ± 0.005 (acc -0.2%)<br>RSD 0.5%	10.4 ± 0.2 (acc +3.9%)<br>RSD 1.7%	101.0 ± 1.2 (acc +1.0%)<br>RSD 1.2%
**Demethyleneberberine**	0.092 ± 0.008 (acc -7.7%)<br>RSD 8.4%	1.1 ± 0.1 (acc +8.4%)<br>RSD 9.1%	10.8 ± 0.4 (acc +7.9%)<br>RSD 3.7%	100.6 ± 2.1 (acc +0.6%)<br>RSD 2.1%
**Columbamine**	0.10 ± 0.008 (acc +4.7%)<br>RSD 7.2%	0.94 ± 0.04 (acc -5.7%)<br>RSD 3.8%	10.5 ± 0.4 (acc +5.5%)<br>RSD 4.2%	101.3 ± 0.6 (acc +1.3%)<br>RSD 0.6%
**Jatrorrhizine**	0.090 ± 0.009 (acc -9.7%)<br>RSD 9.7%	1.01 ± 0.06 (acc +0.8%)<br>RSD 5.8%	10.8 ± 0.3 (acc +8.1%)<br>RSD 2.6%	99.7 ± 1.3 (acc -0.3%)<br>RSD 1.3%
**Berberrubine**	0.097 ± 0.003 (acc -3.0%)<br>RSD 2.7%	0.93 ±0.01 (acc -6.9%)<br>RSD 1.5%	10.2 ± 0.1 (acc +2.1%)<br>RSD 0.7%	94.1 ± 3.1 (acc -5.9%)<br>RSD 3.3%
**Dihydroberberine**	0.091 ± 0.009 (acc -9.3%)<br>RSD 10.0%	0.84 ± 0.11 (acc -15.7%)<br>RSD 13.5%	9.9 ± 0.4 (acc -1.2%)<br>RSD 4.2%	93.6 ± 3.2 (acc -6.4%)<br>RSD 3.4%
**Palmatine**	0.098 ± 0.017 (acc -2.0%)<br>RSD 17.0%	0.90 ± 0.03 (acc -10.4%)<br>RSD 3.1%	10.2 ± 0.2 (acc +2.2%)<br>RSD 2.4%	101.0 ± 2.1 (acc +1.0%)<br>RSD 2.1%

Mean values ± SD (n=3) are given, as well as accuracy (acc, % deviation from nominal value), and precision (as % RSD).

INTESTINAL CONTENT SAMPLES

Quantification of all analytes in intestinal contents of pig was based on the internal standard tetrahydropalmatine, except for berberine and palmatine where berberine-d6 was used as the internal standard compound. Thalifendine was quantified using the berberrubine calibration curve and phase II metabolites were quantified relative to the berberine calibration curve, since no analytical reference standards were available for these compounds. A full 3-day validation experiment was performed on middle jejunum intestinal contents, while for the other jejunal intestinal compartments (proximal and distal) a more limited one-day cross-validation experiment was performed. Also for the other intestinal compartments analyzed (duodenum, ileum, cecum, and colon), a more limited one-day validation experiment was performed with evaluation of linearity and within-day accuracy and precision.

Linearity

Matrix-matched calibration curves were prepared in jejunum (middle) intestinal contents on three different analysis days in the concentration range 25 – 5000 ng/g, including the levels 25, 50, 100, 250, 500, 1000, 2500, and 5000 ng/g. Calibration curves were best described by a quadratic calibration model $y= ax^2 + bx + c$, using a $1/x^2$ fit weighting, resulting as such in the lowest goodness-of-fit coefficient (GoF) %.

As can be seen, the coefficient of determination (R^2) was higher than the 0.99 criterion, while the GoF (%) was below the 10% criterion for all components.

Linearity was evaluated in the same way for the other intestinal compartments (duodenum, ileum, cecum, and colon) at one single occasion, with similar and satisfactory validation outcome as well.

Linearity evaluation for berberine and phase I metabolites in pig middle jejunum intestinal contents, concentration range: 25 – 5000 ng/g.

Compound	Evaluation occasion	a	b	c	R^2	GoF (%)
**Berberine**	Day 1	-1.001e-7	0.007858	-0.008903	0.9993	2.1
	Day 2	-3.974e-8	0.007769	-0.019450	0.9990	2.6
	Day 3	1.123e-8	0.007605	-0.003355	0.9992	2.5
**Demethyleneberberine**	Day 1	-4.416e-8	0.002270	-0.003087	0.9995	1.8
	Day 2	-5.730e-8	0.002284	-0.007571	0.9976	4.4
	Day 3	-9.238e-8	0.002379	-0.001959	0.9943	7.6
**Columbamine**	Day 1	-1.392e-8	0.002147	-0.002646	0.9990	2.6
	Day 2	-1.636e-8	0.002175	-0.010370	0.9986	3.1
	Day 3	-1.538e-8	0.002374	-0.002324	0.9940	7.2
**Jatrorrhizine**	Day 1	-3.176e-8	0.002829	-0.003700	0.9995	1.9
	Day 2	-5.294e-8	0.002994	-0.015680	0.9995	1.8
	Day 3	-3.927e-8	0.003121	0.000930	0.9951	6.4
**Berberrubine**	Day 1	-1.552e-7	0.007120	-0.025140	0.9990	2.6
	Day 2	-2.086e-7	0.007513	-0.049950	0.9990	2.9
	Day 3	-2.101e-7	0.007788	-0.007354	0.9907	9.0
**Dihydroberberine**	Day 1	-2.415e-8	0.002770	-0.008235	1.0000	1.4
	Day 2	-4.627e-8	0.003134	-0.016420	0.9995	1.9
	Day 3	-8.466e-8	0.003319	-0.008497	0.9987	3.5
**Palmatine**	Day 1	-1.054e-7	0.005863	-0.011480	0.9994	2.0
	Day 2	-7.073e-8	0.005796	-0.028800	0.9980	3.7
	Day 3	-4.810e-8	0.005497	-0.009184	0.9995	2.1

Linearity evaluation for berberine and phase I metabolites in pig duodenum, ileum, cecum, and colon intestinal contents, concentration range: 25 – 5000 ng/g.

Compound	Location	a	b	c	R^2	GoF (%)
**Berberine**	Duodenum	7.110e-9	0.007431	-0.031350	0.9995	1.8
	Ileum	5.960e-8	0.007878	-0.019700	0.9989	2.7
	Cecum	-4.561e-8	0.007890	-0.015070	0.9989	2.8
	Colon	-1.181e-8	0.007878	-0.012330	0.9994	2.1
**Demethyleneberberine**	Duodenum	-5.435e-8	0.002520	-0.007631	0.9959	5.5
	Ileum	-8.359e-8	0.002187	-0.005985	0.9945	6.9
	Cecum	-3.742e-8	0.002161	-0.005978	0.9988	2.9
	Colon	-5.243e-8	0.002192	-0.003497	0.9991	2.7
**Columbamine**	Duodenum	4.511e-8	0.002775	-0.011240	0.9960	5.3
	Ileum	2.340e-8	0.002228	-0.009967	0.9984	3.3
	Cecum	2.053e-8	0.002040	-0.004173	0.9994	2.0
	Colon	-3.153e-9	0.002179	-0.006625	0.9972	4.4
**Jatrorrhizine**	Duodenum	1.564e-8	0.003746	-0.011970	0.9974	4.3
	Ileum	3.548e-9	0.003016	-0.011550	0.9989	2.7
	Cecum	-2.561e-9	0.002792	-0.005285	0.9987	3.0
	Colon	-2.664e-8	0.002965	-0.007191	0.9973	4.3
**Berberrubine**	Duodenum	-2.346e-7	0.009369	-0.056030	0.9940	6.6
	Ileum	-1.463e-7	0.007145	-0.042030	0.9955	5.9
	Cecum	-4.785e-8	0.006738	-0.018310	0.9987	3.0
	Colon	-1.018e-7	0.006846	-0.032890	0.9948	6.0
**Dihydroberberine**	Duodenum	-7.134e-8	0.003743	-0.020630	0.9960	5.3
	Ileum	-6.047e-8	0.002840	-0.014890	0.9949	6.0
	Cecum	-1.272e-8	0.003236	-0.010900	0.9983	3.4
	Colon	-2.300e-8	0.003067	-0.016370	0.9979	3.9
**Palmatine**	Duodenum	-1.869e-8	0.005234	-0.033780	0.9986	3.1
	Ileum	-1.541e-8	0.005446	-0.028070	0.9984	3.3
	Cecum	-1.752e-8	0.005704	-0.013680	0.9980	3.8
	Colon	-3.523e-8	0.005705	-0.021400	0.9983	3.5

Accuracy and precision, and limit of quantification

At each level evaluated for accuracy and precision, 25, 250, and 2500 ng/g, 6 independent measurements were performed at each of three analysis days in pig middle jejunum intestinal contents.

Tolerance for within-day accuracy was -20% to +10% at all levels tested (Heitzman, 1994; EU legislation, 1991, 2003, 2004). Maximum tolerance for within-day precision was concentration dependent: 10% at the 250 and 2500 ng/g levels, and 15% at the 25 ng/g level (VICH GL49, 2016). Criteria were met for within-day accuracy and precision for all compounds at each level. Only for berberrubine, at day 2, a borderline failing result was observed for accuracy: + 11.6% at the 2500 ng/g level.

Also between-day accuracy was in agreement with the above-mentioned criteria for all components at each level tested. Between-day precision criteria were also met, as calculated according to the Horwitz equation: 13.9%, 19.7%, and 27.9% at the 2500, 250, and 25 ng/g level respectively (Heitzman, 1994; EU legislation, 2003, 2004).

In addition, within-day accuracy and precision were also tested at lower levels, 5 and 10 ng/g, each level on 6 independent measurements, where it was observed that accuracy and precision were in agreement with the validation limits at these levels (-30% to +10% and -20% to +10% for accuracy, and 25% and 15% for precision, each resp. at 5 ng/g and 10 ng/g), with the exception of failing results for accuracy at the 5 ng/g level (+11.2% for palmatine, and +18.7% for dihydroberberine). Based on this additional validation experiment, the LOQ could be established at 5 ng/g in pig intestinal contents for all compounds included, with the exception of dihydroberberine, where an LOQ of 10 ng/g was established.

For the other jejunal intestinal compartments (proximal and distal), a limited cross-validation experiment was performed, where 3 replicates in both of these matrices were prepared at the 25, 250, and 2500 ng/g levels. These extracts were quantified using a calibration prepared in middle jejunum intestinal contents matrix. The results for accuracy and precision were within

the validation criteria given above, indicating similar analytical behaviour (extraction recovery and matrix effect) in the different jejunal segments. One borderline failing accuracy result (+12.4%) was observed for berberrubine at the 2500 ng/g level in proximal jejunum.

In the other intestinal compartments (duodenum, ileum, cecum, and colon), within-day accuracy and precision were evaluated, with results in agreement with the above mentioned validation limits. A few borderline failing results were observed for berberrubine for accuracy: duodenum, +12.5% at the 2500 ng/g level, cecum, +13.2% and +11.7% at the 250 ng/g and 2500 ng/g levels respectively, and colon, +12.3% at the 2500 ng/g level.

Within-day and between-day accuracy and precision evaluation for berberine and phase I metabolites in pig intestinal contents (middle jejunum) at 25, 250, and 2500 ng/g levels.

Compound	Evaluation occasion	Level 1: 25 ng/g	Level 2: 250 ng/g	Level 3: 2500 ng/g
**Berberine**	**Day 1**	25.0 ± 0.6 (acc +0.2%)<br>RSD 2.4%	249.8 ± 4.2 (acc -0.1%)<br>RSD 1.7%	2599.5 ± 27.6 (acc +4.0%)<br>RSD 1.1%
	**Day 2**	24.2 ± 0.4 (acc -3.3%)<br>RSD 1.6%	248.6 ± 3.8 (acc -0.6%)<br>RSD 1.5%	2531.2 ± 29.2 (acc +1.2%)<br>RSD 1.2%
	**Day 3**	24.3 ± 0.8 (acc -2.8%)<br>RSD 3.4%	253.6 ± 7.0 (acc +1.4%)<br>RSD 2.7%	2532.5 ± 69.4 (acc +1.3%)<br>RSD 2.7%
	**Between-day**	24.5 ± 0.7 (acc -2.0%)<br>RSD 2.9%	250.7 ± 5.3 (acc +0.3%)<br>RSD 2.1%	2554.4 ± 54.5 (acc +2.2%)<br>RSD 2.1%
**Demethyleneberberine**	**Day 1**	25.5 ± 0.6 (acc +2.1%)<br>RSD 2.5%	248.1 ± 3.1 (acc -0.8%)<br>RSD 1.2%	2550.9 ± 19.7 (acc +2.0%)<br>RSD 0.8%
	**Day 2**	25.8 ± 0.7 (acc +3.3%)<br>RSD 2.6%	253.7 ± 3.5 (acc +1.5%)<br>RSD 1.4%	2685.7 ± 31.0 (acc +7.4%)<br>RSD 1.2%
	**Day 3**	25.3 ± 0.7 (acc +1.4%)<br>RSD 2.7%	257.3 ±2.8 (acc +2.9%)<br>RSD 1.1%	2691.0 ± 61.8 (acc +7.6%)<br>RSD 2.3%
	**Between-day**	25.6 ± 0.7 (acc +2.3%)<br>RSD 2.6%	253.0 ± 4.9 (acc +1.2%)<br>RSD 1.9%	2642.5 ± 77.3 (acc +5.7%)<br>RSD 2.9%
**Columbamine**	**Day 1**	25.0 ± 0.3 (acc -0.0%)<br>RSD 1.2%	249.9 ± 5.3 (acc -0.0%)<br>RSD 2.1%	2564.3 ± 19.6 (acc +2.6%)<br>RSD 0.8%
	**Day 2**	26.2 ± 0.5 (acc +4.9%)<br>RSD 1.8%	265.0 ± 8.2 (acc +6.0%)<br>RSD 3.1%	2712.6 ± 58.0 (acc +8.5%)<br>RSD 2.1%
	**Day 3**	24.3 ± 1.0 (acc -2.9%)<br>RSD 4.0%	250.8 ± 3.4 (acc +0.3%)<br>RSD 1.3%	2519.9 ± 68.5 (acc +0.8%)<br>RSD 2.7%
	**Between-day**	25.2 ± 1.0 (acc +0.7%)<br>RSD 4.1%	255.2 ± 9.0 (acc +2.1%)<br>RSD 3.5%	2598.9 ± 98.3 (acc +4.0%)<br>RSD 3.8%
**Jatrorrhizine**	**Day 1**	24.8 ± 1.0 (acc -0.6%)<br>RSD 3.9%	251.4 ± 3.4 (acc +0.5%)<br>RSD 1.4%	2564.8 ± 31.1 (acc +2.6%)<br>RSD 1.2%
	**Day 2**	26.6 ± 0.4 (acc +6.6%)<br>RSD 1.6%	258.7 ± 5.5 (acc +3.5%)<br>RSD 2.1%	2632.4 ± 49.0 (acc +5.3%)<br>RSD 1.9%
	**Day 3**	24.3 ± 0.9 (acc -2.9%)<br>RSD 3.6%	249.5 ± 6.9 (acc -0.2%)<br>RSD 2.8%	2549.4 ± 44.2 (acc +2.0%)<br>RSD 1.7%
	**Between-day**	25.3 ± 1.3 (acc +1.0%)<br>RSD 5.1%	253.2 ± 6.6 (acc +1.3%)<br>RSD 2.6%	2582.2 ± 54.2 (acc +3.3%)<br>RSD 2.1%
**Berberrubine**	**Day 1**	25.9 ± 0.4 (acc +3.7%)<br>RSD 1.5%	251.6 ± 4.3 (acc +0.6%)<br>RSD 1.7%	2620.3 ± 19.9 (acc +4.8%)<br>RSD 0.8%
	**Day 2**	26.7 ± 0.7 (acc +6.8%)<br>RSD 2.6%	262.9 ± 5.7 (acc +5.1%)<br>RSD 2.2%	2790.3 ± 18.2 (acc +11.6%)<br>RSD 0.7%

	**Day 3**	24.1 ± 0.9 (acc -3.7%)<br>RSD 3.6%	251.2 ± 3.6 (acc +0.5%)<br>RSD 1.4%	2738.0 ± 41.5 (acc +9.5%)<br>RSD 1.5%
	**Between-day**	25.6 ± 1.3 (acc +2.3%)<br>RSD 5.1%	255.2 ± 7.1 (acc +2.1%)<br>RSD 2.8%	2716.2 ± 77.9 (acc +8.6%)<br>RSD 2.9%
**Dihydroberberine**	**Day 1**	25.3 ± 0.8 (acc +1.3%)<br>RSD 3.1%	244.8 ± 6.3 (acc -2.1%)<br>RSD 2.6%	2411.3 ± 24.8 (acc -3.5%)<br>RSD 1.0%
	**Day 2**	26.2 ± 0.7 (acc +4.7%)<br>RSD 2.8%	247.5 ± 4.8 (acc -1.0%)<br>RSD 1.9%	2506.0 ± 18.5 (acc +0.2%)<br>RSD 0.7%
	**Day 3**	24.1 ± 0.5 (acc -3.6%)<br>RSD 2.0%	239.1 ± 3.8 (acc -4.4%)<br>RSD 1.6%	2532.9 ± 42.0 (acc +1.3%)<br>RSD 1.7%
	**Between-day**	25.2 ± 1.1 (acc +0.8%)<br>RSD 4.3%	243.8 ± 6.0 (acc -2.5%)<br>RSD 2.5%	2483.4 ± 60.7 (acc -0.7%)<br>RSD 2.4%
**Palmatine**	**Day 1**	24.7 ± 0.7 (acc -1.0%)<br>RSD 2.9%	245.1 ± 4.0 (acc -2.0%)<br>RSD 1.7%	2566.3 ± 29.6 (acc +2.7%)<br>RSD 1.2%
	**Day 2**	24.4 ± 0.6 (acc -2.6%)<br>RSD 2.6%	244.6 ± 4.5 (acc -2.2%)<br>RSD 1.8%	2539.6 ± 20.9 (acc +1.6%)<br>RSD 0.8%
	**Day 3**	23.5 ± 0.6 (acc -5.8%)<br>RSD 2.7%	251.8 ± 6.1 (acc +0.7%)<br>RSD 2.4%	2534.5 ± 71.1 (acc +1.4%)<br>RSD 2.8%
	**Between-day**	24.2 ± 0.8 (acc -3.1%)<br>RSD 3.4%	247.2 ± 5.8 (acc -1.1%)<br>RSD 2.3%	2546.8 ± 45.6 (acc +1.9%)<br>RSD 1.8%

Mean values ± SD (n=6) are given, as well as accuracy (acc, % deviation from nominal value), and precision (as % RSD).

Within-day accuracy and precision evaluation for berberine and phase I metabolites in pig intestinal contents (middle jejunum) at 5, and 10 ng/g levels.

**Compound**	**Level 1: 5 ng/g**	**Level 2: 10 ng/g**
**Berberine**	4.8 ± 0.3 (acc -4.2%)<br>RSD 7.1%	9.5 ± 0.4 (acc -5.0%)<br>RSD 4.2%
**Demethyleneberberine**	5.2 ± 0.2 (acc +4.3%)<br>RSD 3.5%	10.0 ± 0.5 (acc -0.2%)<br>RSD 4.6%
**Columbamine**	5.3 ± 0.2 (acc +5.2%)<br>RSD 4.7%	10.1 ± 0.5 (acc +0.7%)<br>RSD 4.6%
**Jatrorrhizine**	4.2 ± 0.3 (acc -15.1%)<br>RSD 6.8%	9.1 ± 0.3 (acc -8.9%)<br>RSD 3.6%
**Berberrubine**	4.6 ± 0.1 (acc -7.5%)<br>RSD 2.8%	8.8 ± 0.2 (acc -12.5%)<br>RSD 2.8%
**Dihydroberberine**	5.9 ± 0.1 (acc +18.7%)	9.5 ± 0.2 (acc -4.6%)

	RSD 1.2%	RSD 2.1%
**Palmatine**	5.6 ± 0.3 (acc +11.2%)<br>RSD 5.8%	9.7 ± 0.4 (acc -2.9%)<br>RSD 3.9%

Mean values ± SD (n=6) are given, as well as accuracy (acc, % deviation from nominal value), and precision (as % RSD)

Cross-validation experiment for berberine and phase I metabolites in pig intestinal contents (proximal and distal jejunum) at 25, 250, and 2500 ng/g levels.

**PROXIMAL JEJUNUM**			
**Compound**	**Level 1: 25 ng/g**	**Level 2: 250 ng/g**	**Level 3: 2500 ng/g**
**Berberine**	24.3 ± 0.6 (acc -2.8%)<br>RSD 2.6%	245.1 ± 5.0 (acc -2.0%)<br>RSD 2.1%	2498.0 ± 33.3 (acc -0.1%)<br>RSD 1.3%
**Demethyleneberberine**	26.2 ± 0.7 (acc +5.0%)<br>RSD 2.7%	258.9 ± 3.1 (acc +3.6%)<br>RSD 1.2%	2719.3 ± 41.3 (acc +8.8%)<br>RSD 1.5%
**Columbamine**	25.3 ± 0.7 (acc +1.1%)<br>RSD 2.9%	258.5 ± 5.7 (acc +3.4%)<br>RSD 2.2%	2683.7 ± 32.2 (acc +7.3%)<br>RSD 1.2%
**Jatrorrhizine**	24.5 ± 0.8 (acc -2.1%)<br>RSD 3.3%	269.6 ± 3.2 (acc +7.9%)<br>RSD 1.2%	2640.7 ± 66.0 (acc +5.6%)<br>RSD 2.5%
**Berberrubine**	25.0 ± 0.5 (acc +0.0%)<br>RSD 2.0%	268.2 ± 5.6 (acc +7.3%)<br>RSD 2.1%	2809.8 ± 8.4 (acc +12.4%)<br>RSD 0.3%
**Dihydroberberine**	26.4 ± 0.4 (acc +5.4%)<br>RSD 1.6%	254.1 ± 1.3 (acc +1.6%)<br>RSD 0.5%	2511.8 ± 15.0 (acc +0.5%)<br>RSD 0.6%
**Palmatine**	23.3 ± 0.6 (acc -6.6%)<br>RSD 2.5%	244.9 ± 2.0 (acc -2.0%)<br>RSD 0.8%	2494.9 ± 24.1 (acc -0.2%)<br>RSD 1.0%
**DISTAL JEJUNUM**			
**Compound**	**Level 1: 25 ng/g**	**Level 2: 250 ng/g**	**Level 2: 2500 ng/g**
**Berberine**	25.5 ± 1.3 (acc +2.1%)<br>RSD 4.9%	259.4 ± 6.4 (acc +3.8%)<br>RSD 2.5%	2702.5 ± 21.6 (acc +8.1%)<br>RSD 0.8%
**Demethyleneberberine**	24.5 ± 0.8 (acc -2.0%)<br>RSD 3.1%	241.5 ± 0.4 (acc -3.4%)<br>RSD 0.2%	2527.3 ± 22.3 (acc +1.1%)<br>RSD 0.9%
**Columbamine**	26.1 ± 0.6 (acc +4.4%)<br>RSD 2.3%	260.1 ± 5.9 (acc +4.0%)<br>RSD 2.3%	2688.2 ± 34.5 (acc +6.7%)<br>RSD 1.3%
**Jatrorrhizine**	26.3 ± 0.6 (acc +5.3%)<br>RSD 2.1%	253.7 ± 2.2 (acc +1.5%)<br>RSD 0.9%	2606.8 ± 28.7 (acc +4.3%)<br>RSD 1.1%
**Berberrubine**	25.7 ± 0.7 (acc +2.8%)<br>RSD 2.9%	261.4 ± 4.8 (acc +4.6%)<br>RSD 1.8%	2696.9 ± 43.9 (acc +7.9%)<br>RSD 1.6%
**Dihydroberberine**	22.2 ± 0.2 (acc -11.3%)	210.6 ± 4.2 (acc -15.8%)	2112.4 ± 60.7 (acc -15.5%)

	RSD 0.9%	RSD 2.0%	RSD 2.9%
**Palmatine**	23.4 ± 1.1 (acc -6.5%)<br>RSD 4.8%	242.4 ± 5.1 (acc -3.0%)<br>RSD 2.1%	2582.7 ± 17.2 (acc +3.3%)<br>RSD 0.7%

Mean values ± SD (n=3) are given, as well as accuracy (% deviation from nominal value), and precision (as % RSD); quantified using calibration curve prepared in middle jejunum intestinal contents.

Within-day accuracy and precision evaluation for berberine and phase I metabolites in pig intestinal contents (duodenum, ileum, cecum, and colon) at 25, 250, and 2500 ng/g levels.

Compound	Intestinal compartment	Level 1: 25 ng/g	Level 2: 250 ng/g	Level 3: 2500 ng/g
**Berberine**	Duodenum	25.4 ± 0.9 (acc +1.7%)<br>RSD 3.6%	250.0 ± 2.3 (acc +0.0%)<br>RSD 0.9%	2542.8 ± 12.4 (acc +1.7%)<br>RSD 0.5%
	Ileum	25.2 ± 0.4 (acc +0.7%)<br>RSD 1.5%	254.6 ± 1.8 (acc +1.8%)<br>RSD 0.7%	2562.9 ± 30.7 (acc +2.5%)<br>RSD 1.2%
	Cecum	25.7 ± 1.0 (acc +2.8%)<br>RSD 4.1%	254.5 ± 6.7 (acc +1.8%)<br>RSD 2.6%	2606.1 ± 28.0 (acc +4.2%)<br>RSD 1.1%
	Colon	25.5 ± 0.8 (acc +2.1%)<br>RSD 3.3%	262.4 ± 6.6 (acc +4.9%)<br>RSD 2.5%	2634.2 ± 56.4 (acc +5.4%)<br>RSD 2.1%
**Demethyleneberberine**	Duodenum	26.1 ± 0.7 (acc +4.6%)<br>RSD 2.7%	251.3 ± 8.5 (acc +0.5%)<br>RSD 3.4%	2774.2 ± 69.9 (acc +9.9%)<br>RSD 2.5%
	Ileum	26.1 ± 0.9 (acc +4.5%)<br>RSD 3.4%	244.1 ± 1.0 (acc -2.4%)<br>RSD 0.4%	2582.7 ± 82.1 (acc +3.3%)<br>RSD 3.2%
	Cecum	26.7 ± 0.5 (acc +7.0%)<br>RSD 1.9%	260.6 ± 2.4 (acc +4.2%)<br>RSD 0.9%	2639.9 ± 29.7 (acc +5.6%)<br>RSD 1.1%
	Colon	25.6 ± 0.7 (acc +2.3%)<br>RSD 2.8%	255.9 ± 5.9 (acc +2.4%)<br>RSD 2.3%	2661.1 ± 57.9 (acc +6.4%)<br>RSD 2.2%
**Columbamine**	Duodenum	25.1 ± 0.6 (acc +0.3%)<br>RSD 2.2%	257.7 ± 5.8 (acc +3.1%)<br>RSD 2.3%	2487.6 ± 78.7 (acc -0.5%)<br>RSD 3.2%
	Ileum	26.3 ± 0.2 (acc +5.3%)<br>RSD 0.7%	259.6 ± 5.7 (acc +3.8%)<br>RSD 2.2%	2438.3 ± 57.5 (acc -2.5%)<br>RSD 2.4%
	Cecum	26.6 ± 0.8 (acc +6.4%)<br>RSD 2.8%	271.7 ± 6.1 (acc +8.7%)<br>RSD 2.2%	2631.2 ± 69.1 (acc +5.2%)<br>RSD 2.6%
	Colon	24.9 ± 0.7 (acc -0.6%)<br>RSD 3.0%	264.2 ± 8.6 (acc +5.7%)<br>RSD 3.2%	2551.8 ± 51.7 (acc +2.1%)<br>RSD 2.0%
**Jatrorrhizine**	Duodenum	24.8 ± 1.2 (acc -0.8%)<br>RSD 4.8%	259.0 ± 3.7 (acc +3.6%)<br>RSD 1.4%	2470.7 ± 68.6 (acc -1.2%)<br>RSD 2.8%
	Ileum	26.0 ± 0.7 (acc +4.1%)<br>RSD 2.8%	258.4 ± 4.1 (acc +3.4%)<br>RSD 1.6%	2449.6 ± 62.1 (acc -2.0%)<br>RSD 2.5%

	Cecum	25.7 ± 0.9 (acc +2.7%)<br>RSD 3.5%	264.5 ± 6.1 (acc +5.8%)<br>RSD 2.3%	2591.5 ± 28.4 (acc +3.7%)<br>RSD 1.1%
	Colon	25.3 ± 0.7 (acc +1.0%)<br>RSD 2.9%	265.6 ± 4.2 (acc +6.2%)<br>RSD 1.6%	2578.4 ± 64.0 (acc +3.1%)<br>RSD 2.5%
**Berberrubine**	Duodenum	27.1 ± 1.1 (acc +8.2%)<br>RSD 4.2%	268.9 ± 7.1 (acc +7.6%)<br>RSD 2.6%	2813.4 ± 63.7 (acc +12.5%)<br>RSD 2.3%
	Ileum	26.1 ± 0.6 (acc +4.5%)<br>RSD 2.4%	266.5 ± 2.8 (acc +6.6%)<br>RSD 1.1%	2730.2 ± 63.7 (acc +9.2%)<br>RSD 2.3%
	Cecum	27.1 ± 1.0 (acc +8.3%)<br>RSD 3.6%	283.0 ± 5.7 (acc +13.2%)<br>RSD 2.0%	2791.8 ± 52.7 (acc +11.7%)<br>RSD 1.9%
	Colon	26.8 ± 1.0 (acc +7.4%)<br>RSD 3.7%	274.0 ± 4.9 (acc +9.6%)<br>RSD 1.8%	2808.1 ± 31.6 (acc +12.3%)<br>RSD 1.1%
**Dihydroberberine**	Duodenum	25.0 ± 1.0 (acc +0.1%)<br>RSD 3.9%	246.5 ± 2.2 (acc -1.4%)<br>RSD 0.9%	2625.0 ± 72.3 (acc +5.0%)<br>RSD 2.8%
	Ileum	25.5 ± 1.0 (acc +2.1%)<br>RSD 3.9%	256.4 ± 2.1 (acc +2.5%)<br>RSD 0.8%	2623.4 ± 64.6 (acc +4.9%)<br>RSD 2.5%
	Cecum	26.6 ± 1.1 (acc +6.2%)<br>RSD 4.3%	254.7 ± 5.1 (acc +1.9%)<br>RSD 2.0%	2513.6 ± 49.1 (acc +0.5%)<br>RSD 2.0%
	Colon	27.4 ± 1.4 (acc +9.5%)<br>RSD 5.0%	255.4 ± 3.3 (acc +2.2%)<br>RSD 1.3%	2602.3 ± 28.5 (acc +4.1%)<br>RSD 1.1%
**Palmatine**	Duodenum	24.3 ± 1.0 (acc -2.9%)<br>RSD 4.0%	252.5 ± 4.2 (acc +1.0%)<br>RSD 1.7%	2551.3 ± 18.4 (acc +2.1%)<br>RSD 0.7%
	Ileum	26.3 ± 0.9 (acc +5.3%)<br>RSD 3.4%	258.2 ± 2.3 (acc +3.3%)<br>RSD 0.9%	2588.7 ± 63.8 (acc +3.5%)<br>RSD 2.5%
	Cecum	25.6 ± 1.0 (acc +2.6%)<br>RSD 3.9%	249.7 ± 7.4 (acc -0.1%)<br>RSD 3.0%	2568.2 ± 49.2 (acc +2.7%)<br>RSD 1.9%
	Colon	25.7 ± 0.7 (acc +2.7%)<br>RSD 2.7%	251.6 ± 5.3 (acc +2.1%)<br>RSD 2.1%	2648.5 ± 35.9 (acc +5.9%)<br>RSD 1.4%

Mean values ± SD are given, as well as accuracy (acc, % deviation from nominal value), and precision (as % RSD); n=6 independent replicates were analyzed for cecum and colon intestinal contents, and n=3 replicates for duodenum and ileum intestinal contents.

Specificity and carry-over

The specificity was demonstrated by the analysis of blank pig intestinal contents of all intestinal segments, i.e. without the addition of analytes nor IS compounds. In all of these samples, no peaks of berberine or related metabolites were found. Also peaks of berberine-d6 or tetrahydropalmatine were absent (results not shown). Carry-over was evaluated by the injection of a solvent after the injection of the highest 5000 ng/g calibrator sample prepared in each intestinal compartment contents blank material. No peaks were detected in all of these solvent injections, demonstrating that carry-over was absent at the concentration levels involved (results not shown).

Stability in processed sample extract

At each level evaluated, 25, 250, and 2500 ng/g, 3 replicates of prepared sample extracts were re-injected after a 24 h storage time at 10 °C (in the autosampler compartment), and quantified using a freshly prepared matrix-matched calibration curve.

The criteria for accuracy and precision were met (see section on accuracy and precision for criteria) for all components. A few exceptions have to be noted at the lowest 25 ng/g level evaluated, with borderline failing accuracies for jatrorrhizine, columbamine, and berberrubine, +10.6%, +13.1%, and +19.6%, respectively. No obvious stability problem was observed for dihydroberberine, indicating again that stabilization of this particular unstable compound could be achieved by the addition of ascorbic acid during sample preparation.

Stability of berberine and phase I metabolites in processed intestinal content sample extracts (middle jejunum) at the 25, 250, and 2500 ng/g levels, stored at 10 °C for 24 h.

**Compound**	**Level 1: 25 ng/g**	**Level 2: 250 ng/g**	**Level 3: 2500 ng/g**
**Berberine**	27.0 ± 1.1 (acc +8.1%)<br>RSD 4.1%	251.2 ± 5.7 (acc +0.5%)<br>RSD 2.3%	2565.4 ± 43.0 (acc +2.6%)<br>RSD 1.7%
**Demethyleneberberine**	27.2 ± 0.6 (acc +8.9%)<br>RSD 2.0%	240.9 ± 2.0 (acc -3.6%)<br>RSD 0.8%	2443.5 ± 37.2 (acc -2.3%)<br>RSD 1.5%
**Columbamine**	28.3± 1.3 (acc +13.1%)<br>RSD 4.8%	255.7 ± 3.5 (acc +2.3%)<br>RSD 1.4%	2525.9 ± 78.4 (acc +1.0%)<br>RSD 3.1%
**Jatrorrhizine**	27.6 ± 0.3 (acc +10.6%)<br>RSD 1.1%	250.2 ± 3.6 (acc +0.1%)<br>RSD 1.5%	2495.6 ± 56.5 (acc -0.2%)<br>RSD 2.3%
**Berberrubine**	29.9 ± 1.1 (acc +19.6%)<br>RSD 3.6%	259.8 ± 1.3 (acc +3.9%)<br>RSD 0.5%	2660.1 ± 80.9 (acc +6.4%)<br>RSD 3.0%
**Dihydroberberine**	25.6 ± 0.1 (acc +2.3%)<br>RSD 0.6%	235.2 ± 2.8 (acc -5.9%)<br>RSD 1.2%	2267.1 ± 54.1 (acc -9.3%)<br>RSD 2.4%
**Palmatine**	27.4 ± 0.8 (acc +9.5%)<br>RSD 2.9%	246.1 ± 3.0 (acc -1.5%)<br>RSD 1.2%	2533.0 ± 43.8 (acc +1.3%)<br>RSD 1.7%

Mean values ± SD (n=3) are given, as well as accuracy (acc, % deviation from nominal value), and precision (as % RSD).

REFERENCES

Commission Decision of 14 August 2002 implementing Council Directive 96/23/EC concerning the performance of analytical methods and the interpretation of results, January 2004.
website:https://eur-lex.europa.eu/legal-content/EN/TXT/?uri=CELEX:02002D0657-20040110

Establishment by the European Community of maximum residue limits (MRLs) for residues of veterinary medicinal products in foodstuffs of animal origin. The Rules Governing Medicinal Products in the European Community. Volume VI. Commission of the European Communities, Brussels, Luxembourg, 1991.

ICH guideline M10 on bioanalytical method validation and study sample analysis-EMA/CHMP/ICH/172948/2019, July 2022.
website:https://www.ema.europa.eu/en/documents/scientific-guideline/ich-guideline-m10-bioanalytical-method-validation-step-5_en.pdf

VICH topic GL49: Studies to evaluate the metabolism and residues kinetics of veterinary drugs in human food-producing animals: validation of analytical methods used in residue depletion studies-EMEA/CVMP/VICH/463202/2009, January 2016.
website:https://www.ema.europa.eu/en/documents/scientific-guideline/vich-gl49-studies-evaluate-metabolism-residue-kinetics-veterinary-drugs-food-producing-animals_en.pdf

Volume 8 Notice to Applicants and Note for Guidance: Establishment of MRLs for residues of veterinary medicinal products in foodstuffs of animal origin, June 2003.

website: http://ec.europa.eu/health/files/eudralex/vol-8/pdf/vol8_10-2005_en.pdf
